# Differential evolution and particle swarm optimization against COVID-19

**DOI:** 10.1007/s10462-021-10052-w

**Published:** 2021-08-19

**Authors:** Adam P. Piotrowski, Agnieszka E. Piotrowska

**Affiliations:** 1 Institute of Geophysics, Polish Academy of Sciences, Ks. Janusza 64, 01-452 Warsaw, Poland; 2 Faculty of Polish Studies, University of Warsaw, Krakowskie Przedmiescie 26/28, 00-927 Warsaw, Poland

**Keywords:** Particle swarm optimization, Differential evolution, Swarm intelligence, Evolutionary computation, Applications, COVID-19

## Abstract

COVID-19 disease, which highly affected global life in 2020, led to a rapid scientific response. Versatile optimization methods found their application in scientific studies related to COVID-19 pandemic. Differential Evolution (DE) and Particle Swarm Optimization (PSO) are two metaheuristics that for over two decades have been widely researched and used in various fields of science. In this paper a survey of DE and PSO applications for problems related with COVID-19 pandemic that were rapidly published in 2020 is presented from two different points of view: 1. practitioners seeking the appropriate method to solve particular problem, 2. experts in metaheuristics that are interested in methodological details, inter comparisons between different methods, and the ways for improvement. The effectiveness and popularity of DE and PSO is analyzed in the context of other metaheuristics used against COVID-19. It is found that in COVID-19 related studies: 1. DE and PSO are most frequently used for calibration of epidemiological models and image-based classification of patients or symptoms, but applications are versatile, even interconnecting the pandemic and humanities; 2. reporting on DE or PSO methodological details is often scarce, and the choices made are not necessarily appropriate for the particular algorithm or problem; 3. mainly the basic variants of DE and PSO that were proposed in the late XX century are applied, and research performed in recent two decades is rather ignored; 4. the number of citations and the availability of codes in various programming languages seems to be the main factors for choosing metaheuristics that are finally used.

## Introduction

During the year 2020 human activities around the globe have been highly affected by the pandemic of SARS-COV-2 virus and related COVID-19 disease. SARS-COV-2 pandemic has severe impact on the health of the human population (McKee and Stucker 2020), the global economy (Fernandes 2020), and the appreciation of the future perspectives (Fetzer et al. 2020). On the other hand, due to some reduction in greenhouse gas emissions and decreasing energy demands, COVID-19 may contribute to mitigation of the future climatic change (Le Quere et al. 2020; Forster et al. 2020) and restoration of environment (Gillingham et al. 2020; Mandal and Pal 2020; Khan et al. 2020).

As COVID-19 pandemic affected almost every kind of human activity, it also triggered a massive response in versatile fields of science (Nowakowska et al. 2020; Haghani et al. 2020). Among mathematical, technical and information-related disciplines the main contribution to the common fight against COVID-19 may be summarized in the famous word “modeling” (Estrada 2020). Due to the recent rapid development of deep learning (LeCun et al. 2015; Goodfellow et al. 2016), the Artificial Intelligence (Bullock et al. 2020; Mei et al. 2020) and Robotics (Yang et al. 2020) become widely used in COVID-19 related research (Arora et al. 2020; Rasheed et al. 2020; Tseng et al. 2020). Artificial Intelligence methods have been applied to various topics related to the ongoing pandemic, such as virus genome analysis (Saqib Nawaz et al. 2021), detecting pneumonia in COVID-19 patients (Harmon et al. 2020; Farhat et al. 2020; Corbacho Abelaira et al. 2021), predicting the numbers of infected people (Ahmad et al. 2020; Rahimi et al. 2021), classification of medical images of COVID-19 patients (Albahri et al. 2020), or sorting out which information on the pandemic is reliable (Rashmid and Wang 2020). Various detailed reviews on deep learning techniques that are currently being applied for COVID-19 diagnostics may be found in Ozsahin et al. (2020), Roberts et al. (2020), Chiroma et al. (2020), Syeda et al. (2020), or Islam et al. (2021). Also, a wide-scale review of predictive models applied against COVID-19 appeared in Weynants et al. (2020).

Numerous models, or more broadly speaking—tasks closely related with COVID-19 pandemic require optimization. Due to their general applicability, global search heuristics such as evolutionary algorithms (EA) and swarm intelligence (SI) methods found numerous applications in combating COVID-19.

For EA and SI, the 1995 was a kind of a milestone year, when two currently the most prominent population-based algorithms were proposed, namely Differential Evolution (DE, Storn and Price 1995) and Particle Swarm Optimization (PSO, Eberhart and Kennedy 1995). Both methods relatively quickly become at the forefront of EA and SI research and applications—see Neri and Tirronen (2010), Das et al. (2016) or Opara and Arabas (2019) for major historical review on DE, and Poli et al. (2007), Bonyadi and Michalewicz (2017a) or Cheng et al. (2018) for a review on PSO. Both methods were also rapidly hybridized in numerous studies (Das et al. 2008; Xin et al. 2012). A number of DE-based variants, especially those being the extensions of JADE version (Zhang and Sanderson 2009) that were developed by step-by-step improvements (Piotrowski and Napiorkowski 2018) become the winners of recent IEEE Competitions in Evolutionary Computation (Tanabe and Fukunaga 2014; Awad et al. 2016a; Brest et al. 2019; Sallam et al. 2020). However, PSO seems to be more widely applied in various fields of science (in ISI Web of Knowledge, Scopus or Google Scholar databases the phrase “particle swarm optimization” is 2–3 times more popular than “differential evolution”), and it may win against DE also in terms of performance when the computational budget (e.g. the number of allowed function calls) is low (Piotrowski et al. 2017). Irrespective of the popularity or inter-comparisons, both DE and PSO families of methods are of competitive importance to the field of metaheuristics. Both DE and PSO algorithms have for years been widely used in papers related to medicine (Abbas 2002; Casciati 2008; Zhang et al. 2013; Baraldi et al. 2018), features selection, or clustering (Das et al. 2006; Suresh et al. 2009; Zorarpaci and Ozel 2016; Sarkar et al. 2016)—topics that are of wide-scale importance during COVID-19 pandemic.

This paper presents a survey of applications of DE and PSO for solving optimization problems related to COVID-19 pandemic in research papers that appeared (at least in preprint version) in 2020—the first year of the global SARS-COV-2 outbreak. The present study has two main goals.

The first and obvious goal is to summarize the current applications of DE and PSO against COVID-19 for researchers that are interested in solving practical problems related with the ongoing pandemic. This should be discussed in a context related more broadly to metaheuristics, and accompanied with some suggestions for the near future.

The second goal is aimed at community interested in the methodology of DE and PSO. SARS-COV-2 pandemic is a new, rapidly developing and global phenomenon, to which researchers could not prepare in advance. The paper aims at studying how practical users of metaheuristics, when in a hurry, make their choices regarding the specific variant they use, and how they set the research details with respect to methods they use. When we know the problem that is to be solved, some questions are quite obvious: whether numerical or combinatorial, single or multi-objective, dynamic or static methods are needed? Others are, however, more intricate, and may sound too technical for practitioners from many fields of science. For example, explorative or exploitative behaviour of algorithms under consideration should be properly chosen to the problem type (Crepinsek et al. 2013; Kerschke et al. 2019). The number of function calls allowed to be used by optimizer also needs to be appropriate, as it often highly affect the choice of the final solutions (Piotrowski et al. 2017; Price et al. 2019). Of similar importance is the setting of population size (Eiben et al. 1999; Piotrowski et al. 2020) and other control parameters (Clerc and Kennedy 2002; Zaharie 2009), which may be (and often are—in modern variants of DE or PSO) made adaptive in various ways (Brest et al. 2006; Tanabe and Fukunaga 2014). The choice of the optimizer may also depend on various assumed criteria (e.g. Mersmann et al. 2015), or on statistical tests used (Vecek et al. 2014; Derrac et al. 2014; Carrasco et al. 2020). These issues are important for the quality of the COVID-19-related research, because the way they are tackled may highly affect the performance of the solutions found by the optimizers. The present paper is also focused on finding out to what extend the choices of specific optimization algorithms made by researchers combating COVID-19 are guided by the recent EA and SI studies, and whether they are based on the outcomes of some Competitions on Evolutionary Optimization held from year to year, code availability, citations or other factors that are expected to impact popularity. By knowing that, readers may learn to what extent the research performed by DE or PSO community is recognized, and how does it contribute to the most important and rapidly developing scientific directions, of which studying, understanding, preventing and mitigating the SARS-COV-2 pandemic is an ongoing example.

The present research is purely literature-based and is done during the hot period in combating the global COVID-19 pandemic. As a result, it does not offer any new methodology, and cannot claim to be complete even at the time of sending for the review. This review is limited to studies that appeared rapidly in the year 2020, during the hot and somehow chaotic debate on COVID-19 pandemic and its impact on human global activities. To some extent it is based on not-yet-reviewed preprints that were available to the public in 2020. Nonetheless, summarizing the main directions of research against COVID-19 in which DE and PSO algorithms are applied, summing up methodological aspects used in various studies, and sharing opinion on the way they are tackled by practitioners could help preparing the future research, and may also be a useful information for people working everyday on DE and PSO on how their work affect and is recognized by other major scientific disciplines.

Although the main goals of the present survey are restricted to DE and PSO methods, applications of other metaheuristics against COVID-19 are also discussed. However, due to the sheer number of metaheuristic names (just to mention “from ants to whales”, Fausto et al. 2020) and difficulties in finding proper relations between them (Sorensen 2015), writing a paper on metaheuristics in general with respect to such a hot topic as COVID-19 is rather impossible. How quickly the community interested in metaheuristics is able to respond to apparent new kind of inspiration is easily confirmed by SARS-COV-2 itself—despite the virus appeared in 2019, in 2020 already three COVID-19-inspired optimization algorithms have been proposed in the literature (Hosseini et al. 2020; Martinez-Alvarez et al. 2020; Al-Betar et al. 2020).

The next section focuses on the first goal of this paper, namely reviewing and summarizing the main applications of DE and PSO in studies aiming at different aspects of COVID-19-related research. It is determined which metaheuristics are more frequently used, and an attempt to give a reason for their popularity is performed. The third section includes more methodological discussion, related with the choice and settings of DE and PSO algorithms. It includes opinions on the practical implications of choices and settings made in different COVID-19 related studies. In the fourth section the main findings from the study are summarized, and—inevitably subjective—opinions are given on how the recent research on DE and PSO affected the research against COVID-19.

## Applications of differential evolution and particle swarm optimization against COVID-19

**Fig. 1:**
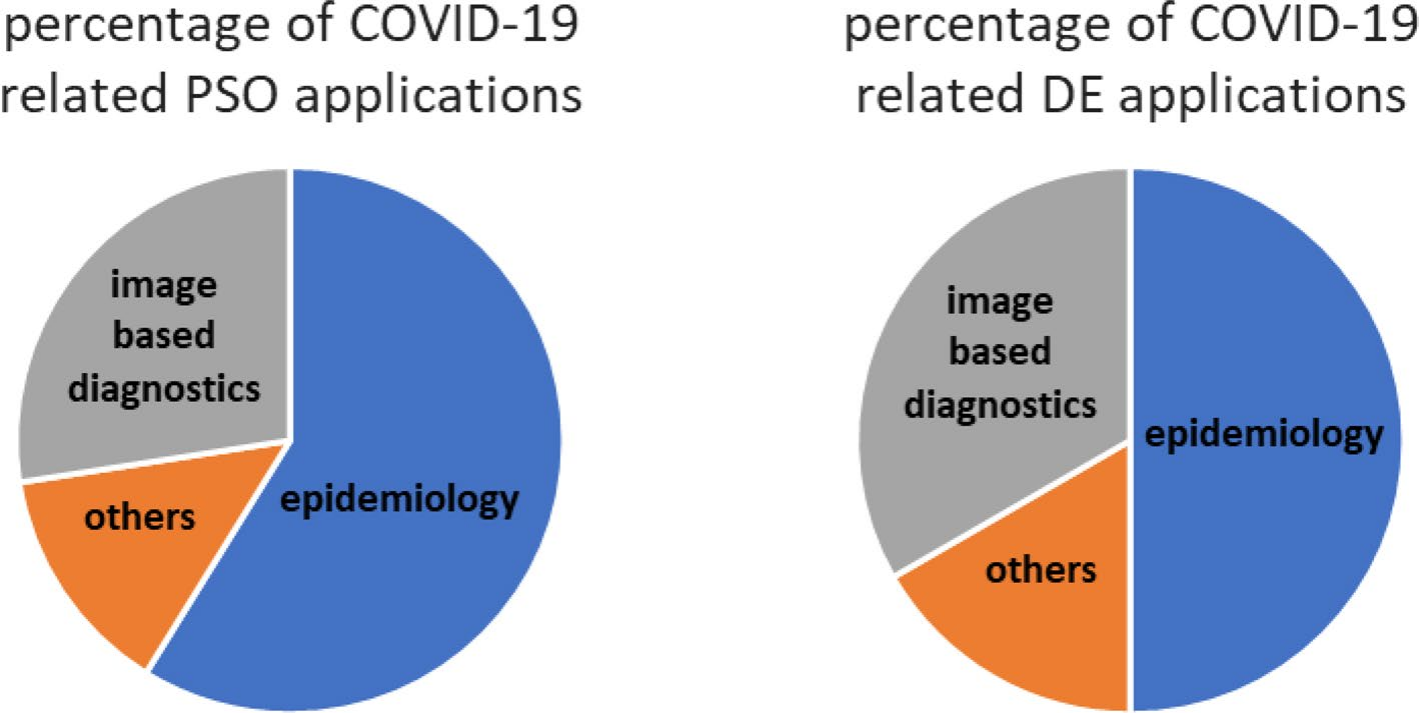
Percentage of the type of COVID-19 related Particle Swarm Optimization (PSO) and Differential Evolution (DE) applications (based on papers included in Tables 1 and 3)

The main areas of research related to SARS-COV-2 virus include pathogenesis, epidemiology, patient diagnostics and treatment (Li et al. 2020c), drug and vaccine development (Jeyanathan et al. 2020), distribution and management of goods or medical equipment (Haghani et al. 2020), and modeling of the effects of government actions (Cheng et al. 2020a). Among these fields, DE and PSO algorithms were mainly used during 2020 in the research on epidemiology, patient diagnostics and goods or equipment management (see Fig. 1).

The details on applications of DE and PSO algorithms against COVID-19 that were available to the public in 2020 are given in Tables 1, 2 and 3 (all papers in Tables 1, 2 and 3 with reference to the year 2021 were available in 2020 at least in preprints). Table 1 contains applications of DE algorithms, Table 2—DE-based Markov Chain Monte Carlo (MCMC) variants (Ter Braak 2006; Vrugt et al. 2009), and Table 3—applications of PSO. In addition, some applications of other metaheuristics related to COVID-19 are given in Table 4. Studies in which both DE and PSO are applied are listed in Table 1, and are not repeated in Table 3. Table 4 contains only studies in which neither DE nor PSO were tackled. In Tables 1, 2, 3 and 4 various details on each application are given. In the column “topic” the main purpose of the particular paper (epidemiology; in host modeling, etc.) is specified, and the reference is given in the column “paper”. The column called “problems/models” specifies either the problem (feature selection, vaccine management, etc.) or the model (SEIR, convolutional neural network, etc.) that is to be optimized by DE, PSO, or other metaheuristics. The subsequent columns contain information on some main properties of the problem that is solved (dimensionality, number of objectives), the metaheuristic algorithms that are used, the main properties of the application (number of runs by each metaheuristic, number of allowed function calls), and the specific information regarding the population size and other control parameters of the algorithms used. If provided in the study, the comparison between different metaheuristics is summarized in the “comparison of performance” column. Depending on the content of particular paper, in that column the methods are either ranked from the best to the worst, or some opinion from the authors are referred (if it is available, but the precise results are not), or reader’s impression of the comparison is given (if authors did not provide a clear statement on which approach performed best or worst). Finally, in the last column some additional comments on DE/PSO applications are given, if necessary. If some information is lacking (or authors of this survey are unable to extract it from the text), the mark “?” is set in the particular column. If “?” is accompanied to specific numbers, it means that the values provided have been assessed by the authors of this review based on the paper content, and hence may be an effect of misunderstanding. It must be reminded here that studies covered in this review have been written by various researchers that represent very different fields of science, and were published in various kinds of journals/ proceedings or were at the time of writing available only in yet un-reviewed preprint versions. As a result, the clarity of details regarding the application of DE/PSO and the effects of their use do vary significantly from paper to paper, and in some cases may be hard to follow. This is why so often “?” mark appears in Tables 1, 2, 3 and 4. However, the information on what is lacking, or unclear, is not less important for the discussion on DE/PSO applicability, as it shows what is considered to be of little interest in particular field of science, or which details seems to be too technical to practitioners (especially when in a hurry during the global pandemic), even if they are of uttermost importance to researchers working on EA or SI methods.

**Table 1:** Differential evolution algorithms against COVID-19

Topic	Paper	Problems/models	Dimensionality	Number of objectives	Algorithms used	Objective function	Number of runs	Number of function calls	Population size	Other control parameters	Comparison of performance	Comments
In host modeling of COVID-19	Abuin et al. (2020)	Ordinary differential equations	3 (?)	1	DE (Storn and Price, 1997)	RMSlogE	?	?	?	?	No	The same model as in Hernandez-Vargas and Velasco-Hernandez (2020). Dimensionality not clearly stated by the authors
In host modeling of COVID-19	Hernandez-Vargas and Velasco-Hernandez (2020)	Ordinary differential equations	3 (?)	1	DE (Storn and Price, 1997)	RMSlogE	?	?	?	?	No	The same model as in Abuin et al (2020). Dimensionality not clearly stated by the authors. DE was chosen following earlier work (Hernandez-Vargas et al, 2014) on influenza virus
Epidemiology and management	Ames et al. (2020)	1. SIR 2. SIHRD	5 (SIR) 10 (SIHRD)	?	1. DE (?) 2. CMA-ES (?) 3. NSGA-II (?)	Specified	?	160.000 (CMA-ES for SIHRD)	400	?	Only CMA-ES results are discussed, authors are satisfied	No references to algorithms used. No detailed information on the number of function calls for SIR or non-CMA-ES algorithms
Epidemiology	Anand et al. (2020)	SIQR + testing	2	1	DE (Storn and Price, 1997)	MSE	?	?	?	?	No	
Epidemiology	de Camino Beck (2020)	SEIRC	2	1	DE with gradient descend (?)	?	?	?	?	?	No	No reference to the algorithm used
Epidemiology	Comunian et al. (2020)	SIR	5	1	DE (Storn and Price, 1997)	Specified	10	3.312 – 31.566 depending on the SIR variant	Default settings (no details)	Default settings (no details)	“Results obtained were very good” (Comunian et al. 2020)	Different SIR variants were tested. Very similar study to Giudici et al. (2020)
Epidemiology	de Falco et al. (2020)	SIR + distancing	3	1	DE (Storn and Price, 1997)	RMSE	1	50.000	50	F = 0.7 CR = 0.9 rand/1/bin	No	
Epidemiology	Fanelli and Piazza (2020)	SIRD	4 or 6	1	DE (Storn and Price, 1997)	?	30	?	?	?	No	Dimensionality depends on the application to the specific country
Epidemiology	Freitas Reis et al. (2020)	SEIR	10	1	DE (Storn and Price, 1997)	Specified	?	?	?	?	No	
Epidemiology	Giudici et al. (2020)	SIRD	5	1	DE (Storn and Price, 1997)	?	10	?	Default settings (no details)	Default settings (no details)	Algorithm “yielded good results”	Very similar study to Comunian et al. (2020)
Epidemiology	Godreev et al. (2020)	SEIRD	6	1	DE (Storn and Price, 1997)	RMSE	?	?	?	?	No	
Epidemiology	Krivorot’ko et al. (2020)	1. SEIR-HZD 2. SEIR-D	8 (SEIR-HZD) 9 (SEIR-D)	1	SEIRHZD: DE (?) SEIR-D: 1. DE (?) 2. SA (?) 3. GA (?) 4. PSO (?)	Specified	?	?	?	?	Although four algorithms were applied, their results were finally not compared	In the paper it is stated that the codes of all algorithms from Python library were used. However, the references to the specific scientific papers are lacking
Epidemiology	Lobato et al. (2020)	SIRD	4	1. SIRD 2. minimization of SIRD maximization of noise	1 objective: 1. DE (Storn and Price 1997) 2. SFS (Salimi 2015) 3. GA (Holland, 1975) 4. FA (Yang 2008) 2 objectives: 1. MODE (Souza et al., (2015) 2. MOSFS (Lobato et al., 2020) 3. NSGA-II (Deb et al., 2002) 4. MOFA (Lobato and Steffen, 2013)	1 objective: scaled MSE 2 objectives: scaled MSE and noise maximization	20	6.250	25	DE/MODE: F = 0.9 CR = 0.9 GA/NSGA-II: CR = 0.8 mutation = 0.01 FA/ MOFA: absorption = 0.9 attractivness = 0.9 SFS: authors refer to Salimi (2015) no information on other specific multi-objective parameters	Marginal differences between single objective algorithms: SFS and FA perform equally, DE worse by 0.001, GA worse by 0.002. No measure for comparison of bi-objective algorithms is given	
Epidemiology	Quaranta et al. (2020)	SAIRD	5	1	DE (?)	Normalized MSE	?	1.500	30	F = 0.9 CR = 0.5 current-to-best/1	No	
Epidemiology	Rica and Ruz (2020)	SIR	5	1	DE (Storn and Price, 1997)	MSE	?	15.000	15	F—sampled for each generation from [0.5,1.0] CR = 0.7	Comparison only with random search	Detailed discussion of the SIR parameters obtained
Epidemiology	Ricardo and Hernandez-Vargas (2020)	SEIR	3	1	DE (Storn and Price, 1997)	RMSE	3.000	?	?	?	No	
Epidemiology and management	Libotte et al. (2020)	1. SIR 2. vaccine management (VM)	3 (SIR) 9 (VM)	1 (SIR) 1–2 (VM)	SIR: DE (Storn and Price, (1997) VM: MODE (Lobato and Steffen, 2011)	SIR: scaled MSE VM: minimizing infected population and number of vaccines used	20	SIR and 1-obj. VM: 2500 2 objective VM: 5000	SIR and 1-obj. VM: 25 2-obj. VM: 50	DE and MODE: F = 0.8 CR = 0.8 rand/1/bin	No	There are 3 applications: 1. DE is used to optimize 3 SIR parameters; 2. DE is used to optimize vaccine use within 9 periods to minimize the number of infections; 3. MODE is used to optimize vaccine use within 9 periods to minimize the number of infections and the number of vaccines provided
Epidemiology	Saif et al. (2021)	ANFIS for predicting the number of COVID-19 cases	?	1	1. DE (?) 2. PSO (?) 3. mutation BA (Saif et al., 2021) 4. GA (?) 5. FA (?) 6. HS (?) 7. TLBO (?) 8. BA (Pham et al., 2005)	RMSE	10	5.000	25 (all algorithms)	DE: F = 0.9 CR = 0.2 PSO: c1 = 2 c2 = 2 w = 1 specified also for other algorithms	Results for India 1. mutation BA 2. PSO 3. BA 4. FA 5. TLBO 6. HS 7. DE 8. GA results for USA: 1. mutation BA 2. BA 3. PSO 4. FA 5. HS 6. TLBO 7. GA 8. DE	The specific variants of algorithms used are undefined, with exception of Bees Algorithm-based ones
Epidemiology	Sanche et al. (2020)	Finding delays between infection and symptoms; modelling the spread of COVID-19 disease to various provinces of China	?	1	DE (Storn and Price, 1995)	Maximization of likelihood	?	?	?	?	No	
Epidemiology and management	Sainz-Pardo and Valero (2020)	Optimal allocation in space and time of COVID-19 infection tests based on SIR-kind of population epidemiology model	“Large number of parameters”	1	DE with directional information (Iorio and Li, 2006)	Specified	?	1.000 iterations (unclear number of function calls)	5	F generated randomly from [0,1] in each generation No crossover	No	Authors considered various numbers of COVID-19 tests, from 10.000 to 500.000. The number of saved infections by optimal allocation of tests is modeled with respect to the homogenous testing in time and space
Human immunological response to COVID-19	Xavier et al. (2020)	Model based on five ordinary differential equations	11	1	DE (Storn and Price, 1997) with constraints handling proposed by Lampinen (2002)	Specified	1 (?)	?	?	?	No	The details of constraint handling approach not specified
Molecular docking	Bhaliya and Shah (2020)	Molegro Virtual Docker	?	1	Guided DE (Thomsen and Christensen, 2006)	?	10	?	?	? (also no information in Thomsen and Christiansen 2006)	No	DE is used to dock molecules with the virus within MVD program
Molecular docking	de Castro et al. (2020)	Molegro Virtual Docker	?	1	Guided DE (Thomsen and Christensen, 2006)	Specified	?	?	?	? (also no information in Thompsen and Christiansen 2006)	No	DE is used to dock molecules with the virus within MVD program
Molecular docking	Sheybani et al. (2020)	Molegro Virtual Docker	?	1	Guided DE (?)	?	10	?	?	?	No	No reference to Guided DE
Molecular docking	Gonzalez-Paz et al. (2020)	Molegro Virtual Docker	?	1	Guided DE (Thomsen and Christensen, 2006)	?	25	?	?	?	No	DE is used within MVD for drugs development
x-ray image diagnostics	Abdel-Basset et al. (2020c)	x-ray image segmentation	Threshold levels 2–30	1	1. iL-SHADE (Brest et al., 2016) 2. HSMA-WOA (Abdel-Basset et al. (2020c) 3. FA (Erdmann et al., 2015) 4. WOA (Abd Elaziz et al., 2017) 5. SSA (Wang et al., 2020b) 6. HHA (Bao et al., 2019) 7. SMA (?)	Specified	20	4.500	30 (for all algorithms)	No information on control parameters of algorithms other than HSMA-WOA and SMA	1. HSMA-WOA 2. SMA 3. WOA 4. HHA 5. FA 6. SSA 7. iL-SHADE	The population size and the number of function calls highly inappropriate for iL-SHADE. It is unclear whether the linear population size reduction is used or not for iL-SHADE. It is also unclear how iL-SHADE was applied to topics like image segmentation
x-ray image diagnostics	Abd Elaziz et al. (2020a)	Feature selection for x-ray chest images	961 (?)	1 combinatorial	1. MRF-DE (Abd Elaziz et al., 2020a) 2. MRF (Zhao et al., 2020) 3. SCA (?) 4. GWO (?) 5. HGS (?) 6. WOA (?) 7. HHO (?)	Accuracy measure	? (but more than 1)	? (only evaluation time is given)	?	?	Averaged over 2 data sets: 1. MRF-DE 2. MRF 3. GWO 4. SCA 5. WOA 6. HGS 7. HHO	Original DE only hybridized with Manta Ray Foraging. Optimizers are used to choose features among those extracted from x-chest images by Fractional Multichannel Exponent Moments. These features are than used by classifier
COVID-19 radiographs	Nowakova et al. (2020)	Column subset selection in matrixes	?	1	DE (Storn and Price, 1997)	Specified	51	40.000	20	F = 0.9 CR = 0.9	No	
COVID-19 patient classification based on tommography chest images	Singh et al. (2020a)	Hyperparameters of CNN	10 (mix of numerical and combinatorial variables)	1	DE (Storn and Price, 1997)	Specified	?	8.000	40	F = 0.1 CR = 0.5	No	DE is claimed to be multiobjective, but two objectives are de facto summed into a single objective problem
COVID-19 patient classification based on tommography chest images	Singh et al. (2020b)	Hyperparameters of CNN	10 (mix of numerical and combinatorial variables)	2	1. MODE (Babu et al., 2005) 2. PSO (?) 3. GA (?)	Specified	?	1.500 (MODE) unclear for PSO and GA	50 (MODE) unclear for PSO and GA	JADE-based mutation and adaptation of F and CR	Unclear	It is claimed that MODE (Babu et al., 2005) is used, but mutation and F, CR adaptation are different in this paper than in Babu et al. (2005). Variants of PSO and GA are not specified. Very different number of epochs is used by CNN trained by PSO, GA and MODE
COVID-19 patient classification based on computer tomography	Punitha et al. (2020)	Feature selection and classification	?	1	1. DE (?) 2. PSO (?) 3. GA (?) 4. DRF (?)	Classification accuracy	10	?	?	?	1. GA 2. DE 3. PSO 4. DRF	In the paper GA is mainly used, other algorithms are just mentioned as competitive methods, without any details. The precise role of metaheuristics used is not given
Impact of environmental factors on COVID-19 cases	Haghshenas et al. (2020)	MLP-ANN	?	1	1. DE (Storn and Price, 1997) 2. PSO (Eberhart and Kennedy, 1995)	MSE	? (probably 1 per case)	450 (used for DE and PSO, various values up to 2.000 are tested)	15 (various values from 5 to 40 are tested)	PSO: c1 = 1.49 c2 = 1.49 w = ? DE: no details	PSO marginally better than DE	DE and PSO were used for MLP-ANN training. No detailed discussion on historical applications is given. No detailed results of various population sizes and numbers of function calls that are said to be tested
Mask production real-time scheduling	Wu et al. (2020)	Large size scheduling instances	?	1	1. SCEA (Zhao et al., 2015) 2. algebraic DE (Santucci et al., 2016) 3.TLBO (Shao et al., 2017) 4. BBO (Du et al., 2018) 5. discrete WWO (Zheng et al., 2019)	Specified	50	100.000	?	?	Averaged from various cases: 1. algebraic DE 2. WWO 3. SCEA 4. TLBO 5. BBO all metaheuristics better than other optimization methods for ANN scheduling	No sufficient details on metaheuristics used. Scheduling problem
COVID-19 prevention programs	Zheng et al. (2020a)	Resources allocation for prevention programs in various communities and resident clustering	? (large)	1 (resident clustering) 1 (with constraints for resources allocation)	For clustering: 1. DE (Storn and Price, 1997) 2. GA (Muhlenbein and Schlierkamp-Voosen, 1993) 3. CLPSO (Liang et al., 2006) 4. hybrid BBO (Ma et al., 2014) 5. EBO (Zheng et al., 2014b) For resources allocation: 1. DE-NM (Luchi and Krohlingb, 2015) 2. WWO (Zheng, 2015) 3. GA (Koziel and Michalewicz, 1999) 4. BBO (Ma and Simon, 2011) 5. improved CS (Abdel-Basset et al., 2018) 6. integer-encoding GWO (Xing et al., 2019)	Specified	30 (for both problems)	?	?	?	For clustering: 1. EBO 2. DE 3. CLPSO 4. hybrid BBO 5. GA For resources allocation: 1. WWO 2. DE-NM 3. CS 4. GWO 5. BBO 6. GA	No sufficient details on compared metaheuristics and allowed number of function calls. A modified version of this study appeared as Zheng et al. (2020c)
COVID-19 resource allocations and costs	Zheng et al. (2020b)	Balancing disease prevention and epidemic control	Main 2-objective problem: 10,000 ~ 40,000 Transformed 2-objective problem: 200 ~ 600	2	Main 2-objective problem 1. MOEA/D (Zhang and Li, 2007) 2. NSGA-II (Deb et al., 2002) 3. CMOEA (Woldesenbet et al., 2009) 4. DECMOSA (Zamuda et al., 2009) 5. D2MOPSO (Al Moubayed et al., 2014) Transformed 2-objective problem: 1. NSGA-II (Deb et al., 2002) 2. MOEA/D 3. DEMOwSA (Zamuda et al., 2007) 4. MOPSO (Zheng et al., 2014a)	Specified	30	? (discussed only for Tabu Search on single-objective sub-problems)	?	?	Comparisons for 14 hospitals are shown; results for transformed problems are much better; depending on the hospital, the best performance is obtained by MOPSO, DEMOwSA or MOEA/D	Discussed algorithms are used to find solutions of 2-objective problems; these are then divided into 1-objective low-dimensional sub-problems that are solved by Tabu search
Goods management during COVID-19 pandemic	Zou et al. (2020)	Goods assignment for supermarkets to address residents needs during pandemics	1000 supermarkets and 6758 communities	2	1. PSO-DE (Zou et al., 2020) 2. ACO (Mouhoub and Wang, 2006) 3. SA (Peng et al., 1996) 4. GA (Ahuja et al., 2000)	Specified; minimization of infection risk and maximization of goods coverage for residents	10	Termination criteria related to pareto front, not the number of function calls	PSO-DE 30 (tested also 10 and 50)	c1 = c2 = 0.005 (also tested 0.1, 0.01, 0.001) w = 0.1 other parameters of PSO-DE hybrid also specified; unspecified for competitors	PSO-DE is considered as the best, as it significantly reduces infection risk, even though its goods coverage efficiency is marginally lower than in the case of other metaheuristics	The references to competing algorithms were not linked to the specific method in the paper; the details of control parameters of competing methods were not specified. However, the sensitivity study for the control parameters of the proposed PSO-DE hybrid is given

Dimensionality refers to the search space in which the algorithm works—if the model has some parameters that are not optimized but fixed/known/assumed by the authors, they are not included in dimensionality. Comparison refers to the comparison between optimization algorithms, not between various models used to solve particular problem. Abbreviations of SIR-based epidemic models: S susceptible, I infected, R recovered, E exposed, C confinement, H hospitalized, Z critical conditions, D deceased, Q quarantined, A asymptomatic, U unrecognized recovered, L lockdown, M migration, N undiagnosed infected. CNN convolutional neural network; MLP-ANN multilayer Perceptron neural network. General abbreviations of metaheuristics (references are given in the Table, as the specific variants do differ): DE differential evolution; PSO particle swarm optimization; GA genetic algorithm; ABC artificial bee colony optimization; BA bees algorithm; BO bowerbird optimizer; BBO biogeography based optimization; CMA-ES covariance matrix adaptation evolutionary strategy; CS cuckoo search; DRF dragonfly algorithm; EBO ecogeography based optimization; EOA equilibrium optimization algorithm; FA firefly algorithm; FPA flower pollination algorithm; GO grasshopper optimization; GSA gravitational search algorithm; GWO grey wolf optimization; HGS henry gas solubility optimization; HHA harris hawks algorithm; HS harmony search; ICA imperialist competitive algorithm; IMA ions motion algorithm; MFA moth-flame algorithm; MPA marine predators algorithm; MRF manta ray foraging; MVO multiverse-optimization; SA simulated annealing; SCA sine cosine algorithm; SCEA shuffled complex evolution algorithm; SFS stochastic fractal search; SMA slime mould algorithm; SSA salp swarm algorithm; SSO spherical search optimization; TLBO teaching learning based optimization; WOA whale optimization algorithm; WWO water wave optimization; MO multi-objective version. ?—if used alone, indicate the lack of information; ?—when follows the text, it means that the information is given but unclear. RMSE root mean square error; MSE mean square error; MAPE mean square percentage error; RMSlogE root mean square logarithmic error

**Table 2:** Differential evolution-based MCMC algorithms against COVID-19

Topic	Paper	Problems/models	Dimensionality	Number of objectives	Algorithms used	Objective function	Number of runs	Number of function calls	Population size	Other control parameters	Comparison of performance	Comments
Epidemiology	Bertuzzo et al. (2020)	SEPIA	7	1	DREAM_ZS_ (Ter Braak and Vrugt, 2008)	?	?	?	?	Partly provided	No	
Epidemiology	Davies et al. (2020)	Deterministic compartmental model	?	1	DE-MCMC (Ter Braak, 2006)	Specified	?	?	?	?	No	
Epidemiology	Gatto et al. (2020)	SEPIA + HQRD	12	1	DREAM_ZS_ (Vrugt et al., 2009)	Specified	?	?	?	Partly provided	No	
Epidemiology	Rahmandad et al. (2020)	Multi-country SEIR	20 (?)	1	DREAM_ZS_ (Vrugt et al., 2009)	Specified	?	1.000.000	?	?	No	
Epidemiology	Wong et al. (2020)	Age of infection model	22 (?)	1	Ensemble of MCMC-DE variants (Ter Braak, 2006; Ter Braak and Vrugt, 2008)	Specified	?	?	?	Partly provided	No	The specific version of the ensemble is undefined

SEPIA epidemiological model refers to: susceptible, exposed, pre-symptomatic, infected with heavy symptoms, asymptomatic/mildly symptomatic. DE differential evolution; MCMC Markov Chain Monte Carlo

**Table 3:** Particle swarm optimization algorithms against COVID-19

Topic	Paper	Problems/models	Dimensionality	Number of objectives	Algorithms used	Objective function	Number of runs	Number of function calls	Population size	Other control parameters	Comparison of performance	Comments
Eoidemiology	Al-Hussein and Tahir (2020)	SEIR	6	1	PSO (?)	Scaled RMSE	?	?	?	?	No	
Epidemiology	Al-qaness et al. (2020a)	ANFIS for infection prediction	?	1	1. MPA (Faramarzi et al. 2020) 2. PSO (?) 3. ABC (?) 4. GA (?) 5. FPA-SSA (Al-qaness et al. 2020b) 6. SCA (?)	MSE	30	2.500	25	PSO: c1 = 2 c2 = 2 w_max_ = 0.9 w_min_ = 0.2 also specified for other algorithms	For USA: 1. MPA 2. PSO 3. GA 4. ABC 5. SCA 6. FPA-SSA for Iran: 1. GA 2. MPA 3. PSO 4. FPA-SSA 5. ABC 6. SCA for Italy: 1. MPA 2. GA 3. PSO 4. SCA 5. FPA-SSA 6. ABC for S. Korea 1. MPA 2. GA 3. FPA-SSA 4. PSO 5. ABC 6. SCA	There are very big differences in root mean square errors between a group of better algorithms (GA, MPA and PSO) and a group of worse algorithms (FPASS, ABC and SCA). It is written that MSE is used as objective function, but results are given for RMSE and other measures
Epidemiology	Al-qaness et al. (2020b)	ANFIS for infection prediction	?	1	1. FPA-SSA (Al-qaness et al. 2020b) 2. PSO (?) 3. GA (?) 4. ABC (?) 5. FPA (Yang 2012)	MSE	30	2.500	25	PSO: c1 = 2 c2 = 2 w_max_ = 0.9 w_min_ = 0.2 also specified for other algorithms	For China: 1. FPA-SSA 2. FPA 3. PSO 4. GA 5. ABC	It is written that MSE is used as objective function, but results are given for RMSE and other measures. Unclear why FPA-SSA perform so poor in Al-qaness et al. (2020a) study
Epidemiology	Al-qaness et al. (2021a)	ANFIS for infection prediction	?	1	1. chaotic MPA (Al-qaness et al. 2021a) 2. PSO (?) 3. MPA (Faramarzi et al. 2020)	RMSE	?	?	?	?	1. chaotic MPA 2. MPA 3. PSO	The model was used for Brazil and Russia, ranking of algorithms is the same in both cases
Epidemiology	Ardabili et al. (2020)	8 simple regression models	1–4	1	1. PSO (?) 2. GA (Whitley et al. 1990) 3. GWO (Mirjalili et al. 2014)	MSE	1 (?)	500.000 (PSO and GWO) 150.000 (GA)	500 (GA and PSO) 1000 (GWO)	?	1. GWO 2. PSO 3. GA	Metaheuristics are used even to fit linear regression model. Different numbers of function calls are used for different methods. Population sizes are very big
Epidemiology	Bowman et al. (2020)	Regression coefficients in 1. Ensemble Model Output Statistics 2. Quantile Regression Averaging	?	1	PSO (Kennedy and Eberhart 1995)	?	?	?	?	?	No	The role of PSO is unclear
Epidemiology	Cordelli et al. (2020)	SIRQ	3	1	PSO (Poli et al. 2007)	Scaled MSE	?	?	?	?	No	
Epidemiology	Dutra et al. (2020)	SIR + unreported symptomatic	3	1	PSO (Kennedy and Eberhart 1995)	Specified	50	?	100	c1 = 2.0 c2 = 2.0 w = 0.9	No	PSO used to select initial solutions for MCMC-particle filter (Liu and West 2001)
Epidemiology	Godio et al. (2020)	SEIR	6	1	HPSO-TVAC (Pace et al. 2019, initially developed by Ratnaweera et al. 2004)	Scaled RMSE	50	30.000	150	?	No	In each run the algorithm converge to almost identical values of 2 SEIR parameters, but very different values for 4 others
Epidemiology	He et al. (2020a)	SEIR	3	1	PSO (Kennedy and Eberhart 1995)	?	?	?	?	?	No	
Epidemiology	He et al. (2020b)	SEIR	2	1	PSO (Kennedy and Eberhart 1995)	Unspecified “error”	1	4.000	40	c1 = 2 c2 = 2 w = after Peng et al. (2019)	No	
Epidemiology	Hoffman (2020)	SEIR	9	1	PSO (Kennedy and Eberhart 1995)	?	?	?	?	?	No	
Epidemiology	Kergassner et al. (2020)	Memory-based spatial infection model	?	1	PSO (Clerc and Kennedy 2002)	Specified	?	?	300	c1 = 1.496172 c2 = 1.496172 w = 0.72984 local topology	No	
Epidemiology	Li et al. (2020a)	?	?	?	?	?	?	?	?	?	?	It is unclear what and how is optimized with PSO
Epidemiology	Makade et al. (2020)	Linear regression (?)	?	?	?	?	?	?	?	?	?	It seems that PSO is used to fit linear regression coefficients
Epidemiology	Naraigh and Byrne (2020)	SEIR	13	1	1. SA (?) 2. PSO (?)	Specified	?	?	?	?	“Results are the same”	No reference to SA or PSO
Epidemiology	Ngie et al. (2020)	Unclear; probably parameter tuning or features selection	?	?	PSO (Kennedy and Eberhart 1995)	?	?	?	?	?	?	
Epidemiology	Niazi et al. (2020)	SNDUR	6	1	PSO (Kennedy and Eberhart 1995)	Specified	?	?	?	?	No	The name of the model has been slightly modified—N is used instead of I, as I already have a different meaning in SIR models discussed in this Table
Epidemiology	Oliveira et al. (2021)	SEIIHURD model	6	1	PSO (Miranda 2018)	Unclear	?	300.000	300	c1 = 0.1 c2 = 0.3 w = 0.9	No	
Epidemiology	Paggi (2020a)	SIRAUL	5 or 7	1	PSO (Kennedy and Eberhart 1995)	Variant of absolute error	?	100.000	100	c1 = 0.5 c2 = 0.5 w_max_ = 0.9 w_min_ = 0.5	No	Dimensionality vary depending on the specific case
Epidemiology	Paggi (2020b)	SIRAD	5	1	PSO (Kennedy and Eberhart 1995)	Variant of absolute error	?	1.000.000	1000	c1 = 0.5 c2 = 0.5 w_max_ = 0.9 w_min_ = 0.5	No	
Epidemiology	Sazvar et al. (2020)	MLP ANN	?	1	1. PSO (Kennedy and Eberhart 1995) 2. GA (Muhlenbein and Mahnig 1999) 3. ICA (Atashpaz-Gargari and Lucas 2007)	MAPE	1 best out of 20	?	?	?	1. GA 2. ICA 3. PSO	ICA and PSO perform very poorly
Epidemiology	Unlu et al. (2020)	SEIR	9	1	PSO (Kennedy and Eberhart 1995)	1-R^2^	1	?	?	?	No	
Epidemiology	Van Tinh (2020a)	Fuzzy logic model	?	1	PSO (Kennedy and Eberhart 1995)	MSE	?	7.500	50	c1 = 2 c2 = 2 w_max_ = 0.9 w_min_ = 0.4	No	Almost the same study as Van Tinh (2020b)
Epidemiology	Van Tinh (2020b)	Fuzzy logic model	?	1	PSO (Kennedy and Eberhart 1995)	MSE	?	3.000	30	c1 = 2 c2 = 2 w_max_ = 0.9 w_min_ = 0.4	No	Almost the same study as Van Tinh (2020a)
Epidemiology	Wang et al. (2020a)	SIR	2	1	PSO (Kennedy and Eberhart 1995)	?	?	?	?	?	No	
Epidemiology	Zhan et al. (2020)	SEIRM	5003 (?)	1	1. PSO (?) 2. GA (?) 3. Pattern Search (?) 4. pseudoevolutionary SA (Zhan et al. 2020)	Specified	?	?	?	?	“These methods cannot provide a satisfied result or cannot even converge in an acceptable computation time (such as one day), while the proposed method can converge to the global optima in two hours”	The paper criticizes the performance of metaheuristics for the particular problem
Epidemiology	Zreiq et al. (2020)	SIR, generalized growth model, classical logistic growth model, generalized logistic model, generalized Richards model	2–4	1	PSO (Kennedy and Eberhart 1995; Boubaker 2017)	MSE	1 (?)	10.000	50	c1 = 2 c2 = 2 w_max_ = 0.9 w_min_ = 0.4	No	Authors refer to Boubaker (2017), but from the text one may infer that they use classical PSO with inertia weight
Epidemiology	Too and Mirjalili (2020)	Selecting features and predicting the fate of a patient	15 features	1	1. binary PSO (Kennedy and Eberhart 1997) 2. HLBDA (Too and Mirjalili 2020) 3. binary DRF (Mirjalili 2016a) 4. binary MVO (Al-Madi et al. 2019)	Specified	20	1.000	10	c1 = 2 c2 = 2 w_max_ = 0.9 w_min_ = 0.4	Very similar accuracy is obtained by all methods, results are only given graphically and it is hard to see any differences; authors claim that HLBDA performed best	The paper aimed mainly at introduction of new metaheuristic (HLBDA) to find an optimal subset of features for classification problems. Tests with COVID-19 disease are added at the end of the paper, after 21 other datasets, and are not discussed in details
Epidemiology and impact of the government interventions on spread of SARS-COV-2 in Brazil	Jorge et al. (2020)	Selected parameters of SEIR model	?	1	PSO (Miranda 2018)	?	?	75.000	150	c1 = 0.1 c2 = 0.3 w = 0.9	No	
Fast Infection Detection	Asghari et al. (2020)	Minimization of bending loss of waveguide	3	?	PSO (?)	?	?	?	?	?	No	In the paper it is just mentioned that for calibration PSO was used
Virus infection detection	Bhonde et al. (2020)	Random forest algorithm for feature detection	?	1	PSO (?)	? (clearly stated for final model only)	?	?	?	c1 = 0.5 c2 = 0.5 w = 0.9 seems to use local topology	No	Role, variant and usage of PSO unclear
Blood test based diagnostics	de Freitas Barbosa et al. (2021)	Feature selection for blood tests	?	1	1. PSO-fs (Wang et al. 2007) 2. Evolutionary search (Kim et al. 2000)	Specified	?	10.000	20	?	Equal performance	
x-ray-based diagnostics	Canayaz (2020)	Feature selection	?	1	1. binary PSO (Too et al. 2019) 2. binary GWO (Too et al. 2018)	Specified	?	2.000	20 (both algorithms)	binary PSO: c1 = 2 c2 = 2 v_max_ = 0.9 w_max_ = 0.9 w_min_ = 0.4 binary GWO unspecified	Binary PSO marginally better than binary GWO	
Computed tomography-based diagnostics	El-Kenawy et al. (2020)	Features selection and classification for CNN (2 distinct problems)	?	1	Feature selection: 1. SFS–Guided WOA (SFS-GWOA, El Kenawy et al. 2020) 2. WOA (Mirjalili and Lewis 2016) 3. GWO (Al-Tashi et al. 2019) 4. GA (Kabir et al. 2011) 5. two-step PSO (Bello et al. 2007) 6. PSO-GWO (Senel et al. 2019) 7. GA-GWO hybrid (?) 8. BA (Karakonstantis and Vlachos 2020) 9. BBO (Simon 2008) 10. MVO (Mirjalili et al. 2016a) 11. BO (Moosavi and Bardsiri 2017) 12. FA (Fister et al. 2012) Classification: 1. PSO-GWOA (EL Kenawy et al. 2020) 2. PSO (?) 3. GWO (?) 4. GA (?) 5. WOA (?)	Specified	20	Feature selection: only given for SFS-GWOA = 800 classification: only given for PSO-GWO = 400	Feature selection: only given for SFS-GWOA = 10 classification: 20 for all metaheuristics	Both problems: two-step PSO: c1 = 2 c2 = 2 w_max_ = 0.9 w_min_ = 0.6 also specified for other metaheuristics	Feature selection: 1. SFS-GWOA 2. GWO 3. BBO 4. MVO 5. GA 6. GA-GWO 7. FA 8. WOA 9. two –step PSO 10. PSO-GWO 11. SBO 12. BO classification: 1. PSO-GWOA 2. PSO 3. GWO 4. GA 5. WOA	The number of function calls for non SFS-GWOA algorithms is unclear It is unclear whether the metaheuristics used for classification are the same as for feature selection or not
x-ray chest image based classification	Goel et al. (2020)	CNN hyperparameters optimization	4	1	1. PSO (Kennedy and Eberhart 1995) 2. GWO (Mirjalili et al. 2014) 3. GA (Holland 1992) 4. Pattern Search (PS, Hooke and Jervis 1961) 5. Simmulated Annealing (SA, van Laarhoven and Aarts 1987) 6. WSO (Mirjalili and Levis 2016)	Specified	?	900 (discussed for GWO only)	30 (discussed for GWO only)	?	1. GWO 2. WOA 3. GA 4. SA 5. PSO 6. PS	No comparison rules are given, number of function calls and population size is specified for GWO only
x-ray chest image based classification	Mohammed et al. (2020)	Threshold in x-ray segmentation	1	1	PSO (Eberhart and Kennedy 1995)	Specified	?	?	?	?	No	
x-ray chest image based classification	Sahlol et al. (2020)	CNN-based feature selection	459 and 462 features	1	1. Fractional-order MPA (FO-MPA, Sahlol et al. 2020) 2. BPSO (?) 3. WOA (Mirjalili and Lewis 2016) 4. HGS (Hashim et al. 2019) 5. SCA (?) 6. SMA (Li et al. 2020b) 7. GWO (Mirjalili et al. 2014) 8. HHO (Heidari et al. 2019) 9. GA (?) 10. MPA (Faramarzi et al. 2020)	Specified	25	300	15	No details on Control parameters	According to Table 4 (performance): Dataset 1: 1. FO-MPA 2. SCA 3. GA 4. BPSO 5. WOA 5. MPA 7. GWO 8. SMA 9. HHO 10. HGS Dataset 2: 1. FO-MPA 2. BPSO 3. GA 4. MPA 5. GWO 6. SCA 7. WOA 8. SMA 8. HGS 10. HHO however, these results seems to disagree with Table 3 (results of the feature selection phase based on fitness function) and discussion in the manuscript; the reason is unclear	No references to GA, SCA and BPSO
x-ray image based classification	Asghar et al. (2020)	CNN-based feature selection	1000 features	1	PSO-fs (Indu et al. 2018)	Specified	?	?	?	?	No	The version of PSO proposed for features selection by Indu et al. (2018) was used
Computer tomography based-diagnostics	Abd Elaziz et al. (2020b)	Multilevel thresholding of computer tomography images	Threshold levels 6–19	1	1. MPA-MFA (Abd Elaziz et al. (2020b) 2. PSO (Kennedy and Eberhart 1995) 3. MPA (Faramarzi et al. 2020) 4. HHO (Heidari et al. 2019) 5. CS (Yang and Deb 2009) 6. GWO (Mirjalili et al. 2014) 7. GO (Mirjalili et al. 2018) 8. SSO (Zhao et al. 2019) 9. MFA (Mirjalili 2015)	Specified	30	2.000	20 (all algorithms)	PSO: c1 = 2 c2 = 2 w_max_ = 0.9 w_min_ = 0.2	2 experiments with 2 ways of comparison: overall: 1. MPA-MFO 2. HHO 3. CS 4. SSO 5. PSO 6. MPA 7. GWO 8. MFA 9. GO	
COVID-19 genome sequence	Issa and Abd Elaziz (2020)	Finding the longest common consecutive subsequence via Fragmented Local Aligner Technique	?	1	1. IMA-PSO (Issa and Abd Elaziz 2020) 2. ASCA-PSO (Issa et al. 2018) 3. IMA (Javidy et al. 2015) 4. SCA (Mirjalili, 2016b) 5. greedy IMA (GIMA, Yang et al. 2018) 6. diversity enhanced IMA (DIMA, Pan et al. 2019)	Specified	20	Only number of iterations is given (larger for IMO-PSO than other algorithms)	From 40 to 700, depending on the consecutive subsequence case	IMO-PSO and ASCA-PSO: c1 = 0.5 c2 = 0.5 w = 0.2; ASCA-PSO: a = 2; also specified for SCA, but not for others	1. IMA-PSO 2. ASCA-PSO 3. GIMA 4. SCA 5. DIMA 6. IMA	It seems that IMA-PSO is allowed to perform more function calls than other methods, but it is not clear from the paper
Remote care for COVID-19 patients by means of moving robotic arms with PID controller	Therib et al. (2020)	PID controller optimization	?	?	PSO (Kennedy and Eberhart 1995)	?	?	?	?	?	No	No details on the role of PSO in the system is provided, apart from a general flowchart
Power consumption under COVID-19 pandemic in China	Huang et al. (2021)	Calibration of specific parameters used by Rolling IMSGM(1,1) model	2 (?)	1	PSO (Kennedy and Eberhart 1995)	Specified	?	?	?	?	No	PSO and ACO are applied for calibration of different kind of parameters during Rolling IMSGM(1,1) model implementation. Although the general role of PSO is specified, the details are unclear
Daily electricity demand during COVID-19 pandemic	Lu et al. (2021)	Support Vector Machine calibration	?	2	1. PSO (Kennedy and Eberhart 1995) 2. multi-objective GWO (Mirjalili et al. 2016b) 3. NSGA-II (Deb et al. 2002) 4. WOA (Mirjalili and Lewis 2016)	Specified	?	?	?	?	1. multi-objective GWO 2. WOA 3. PSO 4. NSGA-II	It is not specified how the basic PSO or WOA were implemented for 2-objective problem
User opinion on mobile applications developed for monitoring the spread of COVID-19 among population	Mustopa et al. (2020)	Support Vector Machine calibration for classification of opinions	1364 opinions from users for classification	1	PSO (?)	?	?	?	?	?	No	The exact role of PSO is unspecified
Internet of Things for students distancing	Alrashidi (2020)	Optimizing the student seats allocation in a classroom	10–250 students in 2-dimensional room	1	1. PSO (?) 2. ACO (?) 3. GA (?)	Distance	20	?	?	PSO: c1 = 0.4 c2 = 0.6 w = 0.8 (?) specified also for ACO, but not for GA	10–20 students: 1. ACO 2. PSO 3. GA 40–250 students: 1. PSO 2. ACO 3. GA	There is an error in inertia weight naming, but it seems that it is set to 0.8
Big Data Application for modelling COVID-19 medical compound	Cholissodin et al. (2020)	Unclear	?	?	PSO (?)	?	?	?	?	?	No	The role of PSO and the variant used are unclear
Mobility of US population during pandemics	Kang et al. (2020)	Minimizing difference between estimated and direct mobile phone-based flow of people	2	1	PSO (Kennedy and Eberhart 1995)	RMSE	?	?	?	?	No	
Impact of lockdown on air quality	Al-qaness et al. (2021b)	ANFIS for air quality estimation: fine particulate matter (PM2.5), carbon dioxine (CO2), sulfur dioxine (SO2) and nitrogen dioxine (NO2)	14 (?)	1	1. PSO (Eberhart and Kennedy 1995) 2. SMA (Li et al. 2020b) 3. PSOSMA (Al-qaness et al. 2021b) 4. GA (?) 5. SCA (?) 6. SSA (?)	MSE	30	3.000	30	c1 = 2 c2 = 2 w_max_ = 0.9 w_min_ = 0.2 specified also for other algorithms	PM2.5: 1. PSOSMA 2. SMA 3. PSO 4. GA 5. SSA 7. SCA CO2: 1. PSOSMA 2. PSO 3. SMA 4. GA 5. SSA 6. SCA SO2: 1. PSOSMA 2. SMA 3. GA 4. PSO 5. SSA 6. SCA NO2: 1. PSOSMA 2. PSO 3. SMA 4. GA 5. SSA 6. SCA	The number of ANFIS parameters is not specified. It was estimated based on the figure provided in the paper, but it is unclear if the number of nodes used is the same as given in the figure. The differences in the final comparison between PSOSMA, PSO, SMA and to some extent GA are small, SSA and SCA perform much poorer
Forecasting currency exchange during COVID-19 pandemics	Hakimah and Kurniawan (2020)	Calibration of double exponential smoothing damped trend model	3	1	1. PSO (Kenedy and Eberhart 1995) 2. GA (?)	Mean absolute percentage error	10	?	?	?	1. PSO 2. GA (marginal difference)	The reference to PSO variant is unclear, but from the text one may infer that the original PSO without inertia weight is used. The variant of GA is unspecified
Relationship between words used in COVID-19 research	Fister et al. (2020a)	Association rule text mining in COVID-19 abstracts	?	1	PSO-ARTM (Fister et al. 2020b)	Specified	5 (Fister et al. 2020b)	10.000 (Fister et al. 2020b)	200	c1 = 2 c2 = 2 w = 0.7	No	

Dimensionality refers to the search space in which the algorithm works—if the model has some parameters that are not optimized but fixed/known/assumed by the authors, they are not included in dimensionality. Comparison refers to the comparison between optimization algorithms, not between various models used to solve particular problem. Abbreviations of SIR-based epidemic models: S susceptible, I infected, R recovered, E exposed, C confinement, H hospitalized, Z critical conditions, D deceased, Q quarantined, A asymptomatic, U unrecognized recovered, L lockdown, M migration, N undiagnosed infected. CNN convolutional neural network; MLP-ANN multilayer Perceptron neural network. General abbreviations of metaheuristics (references are given in the Table, as the specific variants do differ): DE differential evolution; PSO particle swarm optimization; GA genetic algorithm; ABC artificial bee colony optimization; BA bees algorithm; BO bowerbird optimizer; BBO biogeography based optimization; CMA-ES covariance matrix adaptation evolutionary strategy; CS cuckoo search; DRF dragonfly algorithm; EBO ecogeography based optimization; EOA equilibrium optimization algorithm; FA firefly algorithm; FPA flower pollination algorithm; GO grasshopper optimization; GSA gravitational search algorithm; GWO grey wolf optimization; HGS henry gas solubility optimization; HHA harris hawks algorithm; HS harmony search; ICA imperialist competitive algorithm; IMA ions motion algorithm; MFA moth-flame algorithm; MPA marine predators algorithm; MRF manta ray foraging; MVO multiverse-optimization; SA simulated annealing; SCA sine cosine algorithm; SCEA shuffled complex evolution algorithm; SFS stochastic fractal search; SMA slime mould algorithm; SSA salp swarm algorithm; SSO spherical search optimization; TLBO teaching learning based optimization; WOA whale optimization algorithm; WWO water wave optimization; MO multi-objective version. ?—if used alone, indicate the lack of information; ?—when follows the text, it means that the information is given but unclear. RMSE root mean square error; MSE mean square error; MAPE mean square percentage error; RMSlogE root mean square logarithmic error. The papers discussed in Table 1 (DE applications) are not repeated here

**Table 4:** Other metaheuristics against COVID-19

Topic	Paper	Problems/models	Dimensionality	Number of objectives	Algorithms used	Objective function	Number of runs	Number of function calls	Population size	Other control parameters	Comparison of performance	Comments
Epidemiology	Pinter et al. (2020)	MLP training to predict the number of infected cases and fatalities	60 84 108 (?)	1	ICA (Atashpaz-Gargari and Lucas 2007)	RMSE (?) (probably, three different criteria are mentioned)	?	Case 1: 12.000 case 2: 13.750 (?)	Case 1: 300 case 2: 250	?	No	In the paper neither the dimensionality nor the number of function calls is explicitly given. Dimensionality is estimated according to the number of MLP nodes; number of function calls is estimated according to data given in the paper
Epidemiology	Yousefpour et al. (2020)	SEIR with government policies	5 (?)	2	GA (?)	Specified	?	50.000	70	Specified	No	Various control parameters are given, but the algorithm is not specified. Dimensionality is not clearly given in the paper
Epidemiology and control	Hadi and Ali (2021)	Controller with use of SEIR	5 (?)	1	Most Valuable Player Algorithm (Bouchekara 2017)	Specified	1	400 (?)	10 (?)	Specified	No	The details of the procedure applied are not clearly explained
Patient diagnostics	Shaban et al. (2020)	Features selection from computer tommography images for classifiers	?	1	GA (Khare and Burse 2016)	Accuracy	?	8 (?)	4 (?)	Specified	No	In Table 5 (Shaban et al. 2020) it is specified that there are 2 generations and the population size is equal to 4
Patient diagnostics and treatment prediction	Elghamrawy and Hassanien (2020)	Features selection for patient classification within AIMDP model	?	1	WOA (Mirjalili and Lewis 2016)	?	?	?	?	?	Unclear	No details, but it is shown that the AIMDP model without WOA-based features selection perform much poorer
x-ray image diagnostics	Abdel-Basset et al. (2020b)	x-ray image segmentation	Threshold levels 10–100	1	1. improved MPA (Abdel-Basset et al. 2020b) 2. MPA (Faramarzi et al. 2020) 3. SCA (Mirjalili 2016b) 4. WOA (Abd Elaziz et al. 2017) 5. EOA (Abdel-Basset et al. 2020a) 6. HHA (Bao et al. 2019) 7. SSA (Wang et al. 2020b)	Specified	20	3.000	20	Unspecified	Average performance from multiple competitions: 1. improved MPA 2. MPA 3. EOA 4. WOA 5. HHA 6. SSA 7. SCA	
x-ray image diagnostics	Altan and Karasu (2020)	Feature matrix coefficients for deep learning	?	1	Chaotic SSA (Sayed et al. 2018)	Specified	?	?	?	?	No	
x–ray image diagnostics	Ezzat et al. (2020)	Hybrid CNN hyperparameters	3	1	GSA (Rashedi et al. 2009)	Specified	?	450	30	?	No	
x-ray image diagnostics	Medjahed and Ouali (2020)	Feature selection for patient classification by different models	844 features	1	Binary version of MVO (Mirjalili et al. 2016a; Medjahed and Ouali 2020)	Specified	?	300	60	Specified	No	
x-ray image diagnostics	Mishra et al. (2020)	CNN weights optimization	?	1	WCA (Qiao et al. 2018)	Specified	?	?	?	?	No	
x-ray image classification	Yousri et al. (2021)	Feature selection from patient images	?	1	1. CS (Yang and Deb 2009) 2. fractional-CS (Yousri and Mirjalili 2020) 3. fractional-CSML (Yosuri et al. 2021) 4. fractional-CSP ( Yosuri et al. 2021) 5. fractional-CSC ( Yosuri et al. 2021) 6. fractional-SCW ( Yosuri et al. 2021) 7. HHA (Heidari et al. 2019) 8. HGS (Hashim et al. 2019) 9. WOA (Mirjalili and Lewis 2016) 10. SSA (Ibrahim et al. 2019) 11. GWO (Ibrahim et al. 2018) 12. SGA (?)	Specified fitness and accuracy	?	750	15	?	Specific ranking of algorithms depends on the criteria used (12 different were presented), but for each case either fractional-CSML, fractional-CSW or fractional-CSC performed best	CSML, CSP, CSC and CSW refer to Cuckoo search that, instead of levy flight, uses Mittag–Leffler (CSML), Pareto (CSP), Cauchy (CSC) or Weibull (CSW) distributions. All these variants were defined in the paper by Yousri et al. (2021). Two different COVID-19 datasets and the best, mean and the worst fitness, as well as the best, mean and the worst accuracy were used for comparison of algorithms (together 12 criteria)
x-ray image classification	Babukarthik et al. (2020)	DCNN architecture	?	1	GA (Babukarthik et al. 2020)	Specified	?	?	?	Discussed	No	Architectures of DCNN are evolved with genetic operators to find the best classifier
x-ray image diagnostics	Vrbancic et al. (2020)	DCNN hyperparameter’s optimization	4	1	GWO for tuning (Vrbancic et al. 2019)	Specified	?	2.500	50	?	No	
Computer tomography based diagnostics	Satapathy et al. (2020)	Thresholding in computer tomography scans	3-level thresholding	1	CS (Yang and Deb 2009)	Specified	?	140.000	40	?	No	
Computer tomography based diagnostics	Yao and Han (2020)	MLP network calibration	?	1	BBO (Ma et al. 2012)	?	?	?	?	?	No	Unclear BBO application
Drug development	Cheng et al. (2020b)	Genetic operations on drug molecules	?	1 with penalty function	Graph-based GA (?)	Specified	?	?	?	?	No	Although not specified clearly in the paper, it seems that GA used is based on Pawar and Bichkar (2015)
Contactless vehicle routing problem during COVID-19 pandemic for food distribution	Chen et al. (2020)	Contactless joint distribution model for food distribution in Wuhan, China	?	1	1. ABC with Tabu search operator and mechanism of progressive construction solution (Chen et al. 2020) 2. enhanced ABC (Szeto et al. 2011) 3. Tabu Search (Glover 1986)	Specified	20	?	?	Discussed	1. ABC with Tabu search 2. Tabu Search 3. enhanced ABC	
Vehicle routing problem during COVID-19 pandemic	Liu et al. (2020)	Model of medical waste transport routes	?	1	Immune ACO with Tabu search (Liu et al. 2020)	Specified	?	?	?	?	No	
Government actions during COVID-19 pandemic	Miralles-Pechuan et al. (2020)	Model daily actions performed by government within SEIR approach	4 possible actions during 200 days = 4^200^ combinations	1	GA (Whitley 1994)	Specified	?	100.000	100	Specified	Reinforcement learning is a better way to determine government actions during pandemic than GA	

Dimensionality refers to the search space in which the algorithm works—if the model has some parameters that are not optimized but fixed/known/assumed by the authors, they are not included in dimensionality. Comparison refers to the comparison between optimization algorithms, not between various models used to solve particular problem. Abbreviations of SIR-based epidemic models: S susceptible, I infected, R recovered, E exposed, C confinement, H hospitalized, Z critical conditions, D deceased, Q quarantined, A asymptomatic, U unrecognized recovered, L lockdown, M migration. CNN convolutional neural network; MLP-ANN multilayer Perceptron neural network. General abbreviations of metaheuristics (references are given in the Table, as the specific variants do differ): DE differential evolution; PSO particle swarm optimization; GA genetic algorithm; ABC artificial bee colony optimization; BA bees algorithm; BO bowerbird optimizer; BBO biogeography based optimization; CMA-ES covariance matrix adaptation evolutionary strategy; CS cuckoo search; DRF dragonfly algorithm; EBO ecogeography based optimization; EOA equilibrium optimization algorithm; FA firefly algorithm; FPA flower pollination algorithm; GO grasshopper optimization; GSA gravitational search algorithm; GWO grey wolf optimization; HGS henry gas solubility optimization; HHA harris hawks algorithm; HS harmony search; ICA imperialist competitive algorithm; IMA ions motion algorithm; MFA moth-flame algorithm; MPA marine predators algorithm; MRF manta ray foraging; MVO multiverse-optimization; SA simulated annealing; SCA sine cosine algorithm; SCEA shuffled complex evolution algorithm; SFS stochastic fractal search; SMA slime mould algorithm; SSA salp swarm algorithm; SSO spherical search optimization; TLBO teaching learning based optimization; WOA whale optimization algorithm; WWO water wave optimization; MO multi-objective version. ?—if used alone, indicate the lack of information; ?—when follows the text, it means that the information is given but unclear. RMSE root mean square error; MSE mean square error; MAPE mean square percentage error; RMSlogE root mean square logarithmic error. Papers discussed in Tables 1 and 3 are not repeated here

### DE and PSO for COVID-19 epidemiological models

From Tables 1 and 3 one may easily note that both DE and PSO algorithms are used against COVID-19 with similar frequency (PSO is slightly more popular than DE) and to solve similar type of optimization problems. Their most frequent application is the calibration of epidemiological models, especially SIR/SEIR ones (see Fig. 1). These are classical, so-called compartmental differential equation models, in which each part of the human population of particular region is included in a kind of compartment like susceptible (S), infected (I), exposed (E), or recovered (R) people (Hethcote 2000).

In the research against COVID-19, DE and PSO algorithms are frequently used to optimize some or all of SIR/SEIR parameters; “some”—as often part of the SIR/SEIR parameters is set empirically, based on literature findings or public/hospital databases (e.g. Oliveira et al. 2021; de Camino Beck 2020; He et al. 2020b). In the case of the basic SIR/SEIR models, the number of calibrated parameters is often limited to 2–6 (Ames et al. 2020; Comunian et al. 2020; Ricardo and Hernandez-Vargas 2020; Al-Hussein and Tahir 2020; Godio et al. 2020; He et al. 2020a, b; Zreiq et al. 2020; Rica and Ruz 2020), but in case of multi-country variants of the model, it may be much larger: Rahmandad et al. (2020) considered 20 parameters to be calibrated, however in that study not a classical DE, but DE-based Markov Chain Monte Carlo (MCMC) sampling approach (Vrugt et al. 2009) was used, and Zhan et al. (2020) considered a distributed SEIRM model with thousands of parameters. Various modified versions of SIR/SEIR models are also being optimized with DE/PSO algorithms. Such modified SIR/SEIR variants often include more kinds of compartments with various additional classes of human population, like those who are hospitalized (H, Ames et al. 2020; Oliveira et al. 2021), deceased (D, Ames et al. 2020; Oliveira et al. 2021; Paggi 2020b; Fanelli and Piazza 2020; Giudici et al. 2020; Godreev et al. 2020; Lobato et al. 2020; Quaranta et al. 2020), quarantined (Q, Cordelli et al. 2020), confined (C, the term is loosely related to quarantined, de Camino Beck 2020), asymptomatic (A, Qaranta et al. 2020; Paggi 2020a), unrecognized recovered (U, Oliveira et al. 2021; Paggi 2020a), in critical conditions (Z, Krivorot’ko et al. 2020), as well as the effects of lockdown (L, Paggi 2020a) or migration (M, Zhan et al. 2020). Such extended variants of SIR/SEIR models often have more parameters for calibration. However, the total number of parameters to be calibrated generally remains lower than 20.

Jorge et al. (2020) showed the impact of government policies on spread of SARS-COV-2 in Brazil in early 2020 using modified SEIR model that was partly calibrated using PSO. Sainz-Pardo and Valero (2020) have shown a bit different study based on SEIR modeling. They analyzed how the proper allocation of thousands of COVID-19 tests in space and time may limit the number of infections in New York state counties. Authors tested variants with different number of available tests and time-varying model parameters. However, the DE variant used was applied with population size set to only 5 and without crossover, what may affect the possibility of finding the optimal solutions and hence impact the final outcome of the study.

In the vast majority of cases when DE/PSO are used to calibrate SIR/SEIR models the problem is single-objective. There are, however, some exceptions. In Lobato et al. (2020) a MODE (Souza et al. 2015) variant and three other multi-objective metaheuristics are used to minimize the mean square error (MSE) of the SIRD model and at the same time maximize the noise within robust optimization framework (Tsutsui and Ghosh 1997). Unfortunately, authors did not comment the quality of the bi-objective solutions found, and did not compare the performance of multi-objective algorithms; when they solve single-objective calibration problem of SIRD, each method perform almost equally well. Libotte et al. (2020) used earlier version of MODE (Lobato and Steffen 2011) to calibrate 3 parameters of SIR model in order to minimize the COVID-19 impact assuming the vaccine is available, and to minimize the number of vaccine dozes used (hence, they solved bi-objective problem). Unfortunately, no comparison against other optimizers is presented.

When DE and PSO are used for single-objective SIR/SEIR problems, the goal is to optimize their parameters, often for a specified country. In the majority of studies there is no comparison against other algorithms, and authors do not express opinion on DE/PSO performance. Some authors commented the quality of solutions obtained, but these studies also rather lack a detailed comparison. Unfortunately, this is frequent in epidemiological papers, even not related to the current pandemic; for example, Cantun-Avila et al. (2021) proposed to use DE for calibration of SEIR model for the epidemic of 2003 SARS virus, but the results were not compared against other methods. With respect to COVID-19 disease, Ames et al. (2020) used DE, CMA-ES and NSGA-II algorithms to calibrate 3-dimensioanl SIR and 5-dimensional SIRHD models; it was unclear why multi-objective NSGA-II was used together with single-objective DE and CMA-ES. None algorithm was backed by a reference, and finally only CMA-ES results were discussed and considered to be appropriate. Comunian et al. (2020) were satisfied with DE (Storn and Price 1997) performance for 5-dimensional SIR calibration. Naraigh and Byrne (2020) used both Simulated Annealing (SA) and PSO (without specifying variants) and found both results to be “the same”. On the contrary, Zhan et al. (2020) considered a distributed version of SIR variant with 300 cities in China and thousands of parameters and found PSO, together with Genetic Algorithms (GA) and Pattern Search (in none case the variant was specified or backed by a reference) to be unable to solve the problem. Authors proposed their own pseudo-evolutionary approach which turned out efficient. Rica and Ruz (2020) found the basic DE a better choice than the random search for the classical SIR model applied to data from Chile.

Some authors found DE/PSO useful for optimization of other kinds of models that are applied to epidemiological research. Saif et al. (2021) used DE, PSO and six other metaheuristics to calibrate ANFIS (Jang 1993) parameters for COVID-19 cases prediction. Tests were performed separately for pandemic data from USA and India; in both cases PSO was among the best methods, but was outperformed by mutation-based Bees Algorithm (proposed in Saif et al. 2021); DE was among two the poorest methods. Unfortunately, the variants of the compared algorithms were neither defined nor referred to, and only from the classical settings of control parameters the reader may infer that the basic versions of DE and PSO were used. Al-quaness et al. (2020a, b, 2021a) performed three similar studies using PSO and 2–5 other metaheuristics (DE was missed in these analyzes) for calibration of ANFIS parameters. Unfortunately, again the variants of PSO and most other metaheuristics were not specified. ANFIS models were calibrated for 7 different countries; for five countries PSO ranked in the middle of the pack, for the remaining two was the poorest; generally Marine Predator Algorithm (MPA, Faramarzi et al. 2020) or GA (unfortunately, unspecified) performed best. Ardabili et al. (2020) used unspecified variant of PSO to calibrate 8 different simple regression models with 1–4 parameters for epidemiological modelling. They found PSO better than GA (Whitley et al. 1990) and poorer than Grey Wolf Optimizer (GWO, Mirjalili et al. 2014); however, it seems that metaheuristics are used even to fit parameters of linear regression models in that study. PSO was also found clearly inferior to GA (Muhlenbein and Mahnig 1999), and poorer than Imperialist Competitive Algorithm (ICA, Atashpaz-Gargari and Lucas 2007) when used to calibrate Multilayer-Perceptron ANN parameters for epidemiological modeling task (Sazvar et al. 2020).

PSO was also used (more frequently than DE) to optimize various other epidemiological models (Bowman et al. 2020; Kergassner et al. 2020; Li et al. 2020a; Ngie et al. 2020; Van Tinh 2020a, b). However, in the majority of these applications information on PSO, or the reason of its use, is very scarce. The exception is Zreiq et al. (2020) paper, in which all details on PSO applied to calibrate 2–4 parameters of each among five different models (including SIR) were given; authors compared performance of the models, bud used only a single calibration method. On the contrary, for calibration of various epidemiological models DE is more frequently used within MCMC framework (Ter Braak 2006; Vrugt et al. 2009, see Table 2). Finally, binary PSO was also tested against four other metaheuristics on feature selection task aimed at prediction of the fate of the patients (Too and Mirjalili 2020). In that study almost equal performance of all algorithms was obtained.

### DE and PSO for image-based COVID-19 diagnostics

Apart from epidemiology, the second most frequent application of DE or PSO algorithms against COVID-19 is x-ray image or computer tomography based diagnostic. In such studies DE or PSO variants are used for feature selection (Abd Elaziz et al. 2020a; Asghar et al. 2020; Canayaz 2020; El Kenawy et al. 2020; Sahlol et al. 2020; Punitha et al. 2020), image segmentation or thresholding (Abdel-Basset et al. 2020c; Abd Elaziz et al. 2020b; Mohammed et al. 2020), or calibration of convolutional ANN (CNN) hyperparameters (Goel et al. 2020; Singh et al. 2020a, b). Like in case of epidemiological models, PSO is slightly more frequently used than DE. These are often non-numerical tasks, and hence may require specific DE and PSO variants (e.g. binary PSO and binary GWO for feature selection in Canayaz 2020). In x-ray and computer tomography imaging applications authors frequently compare more than one metaheuristic for specific problem (Abdel-Basset et al. 2020c, Abd Elaziz et al. 2020a, b; Singh et al. 2020b; Canayaz 2020; El Kenawy et al. 2020; Goel et al. 2020; Sahlol et al. 2020; Punitha et al. 2020). Unfortunately, in the references given in particular paper readers may sometimes find either typical numerical optimizers, or a mix of, e.g. feature selection-oriented and numerical methods (e.g. El-Kenawy et al. 2020; Sahlol et al. 2020). In some papers the variants of specific algorithms are not given at all (Abd Elaziz et al. 2020a; El-Kenawy et al. 2020; Singh et al. 2020b; Sahlol et al. 2020). As a result, although a great effort is made by various researchers to compare different metaheuristics for different goals related to image-based diagnostics of COVID-19 disease, the final outcome must be treated with caution.

DE-based algorithms were already rather rarely compared against other metaheuristics on image-based COVID-19 diagnostic tasks. Abdel-Basset et al. (2020c) found iL-SHADE (Brest et al. 2016) unfit for segmentation of x-ray images. This result is not surprising, considering that iL-SHADE was developed for typical numerical optimization problems, that the population size of all algorithms compared was fixed to 30 (iL-SHADE requires linear decrease of population size from very large number at the early stage of the search to very small number at the end), and that the number of function calls was limited to 4500 (iL-SHADE aims at exploration, hence is efficient when the number of function calls is large). In another study, Abd-Elaziz et al. (2020a) found DE coupled with Manta Ray Foraging algorithm (Zhao et al. 2020) the best among seven metaheuristics for the problem of feature selection of x-ray images. In Punitha et al. (2020), an unspecified DE version was ranked the second best approach, better than (also unspecified) PSO, but much worse than Genetic Algorithm; however, the precise role of the compared metaheuristics is not stated in this study.

PSO algorithms are more frequently used and compared against other metaheuristics for image-based COVID-19 diagnostic, but show similarly uneven performance. Canayaz (2020) found binary PSO slightly better than binary GWO for x-ray image feature selection. El-Kenawy et al. (2020) found that neither two-step PSO variant (Bello et al. 2007) nor PSO and GWO hybrid (Senel et al. 2019) perform well for feature selection from computed tomography images; however, in the same study PSO coupled with GWO performed best for the classification task. PSO also turned out among the poorest methods for CNN hyperparameter optimization (Goel et al. 2020). On the contrary, in Sahlol et al. (2020) an undefined BPSO variant performed relatively well (being 2–4th best method out of 10) on CNN-based feature selection problems. In Abd Elaziz et al. (2020b) PSO finished in the middle of the pack (5th place among 9 metaheuristics) on multilevel thresholding task for computer tomography-based images.

From the analyzed comparisons on x-ray or computer tomography-based diagnostics of COVID-19 disease readers may infer that PSO is a bit more popular than DE, and that depending on the specific problem, DE/PSO variant or data set used, the results may be contradictory. In some applications DE or PSO perform best, in others—are among the worst metaheuristics. Hence, despite the effort made, one cannot find any clue regarding the usefulness of DE or PSO for these particular tasks.

### Other applications of DE and PSO against COVID-19

DE and PSO were also compared against each other, and against other optimizers, in a few other applications against COVID-19. Haghshenas et al. (2020) used both DE (Storn and Price 1997) and PSO (Eberhart and Kennedy 1995) to calibrate Multilayer-Perceptron ANN parameters for searching of environmental factors that may impact the spread of SARS-COV-2 virus; authors did not specify much details on the DE variant used, but found PSO marginally better. According to Zheng et al. (2020a), who was looking for the best resources allocation programs for various communities, the basic DE (Storn and Price 1997) turned out the second best method for clustering of residents problem and, if coupled with Nelder Mead algorithm (Nelder and Mead 1965), the second best for the problem of resources allocation for clustered residents. For the clustering problem DE outperformed CLPSO and four other competitors. Zheng et al. (2020b) also considered optimization of resources allocation for hospitals by studying 2-objective optimization problems, and found that PSO and DE-based algorithms are among the best performing ones for some studied cases; however, the study found that none algorithm may be recommended for all analyzed cases.

In various papers DE found versatile other applications against COVID-19. Abuin et al. (2020) and Hernandez-Vargas and Velasco-Hernandez (2020), in two very similar studies, presented an application of the basic DE algorithm (Storn and Price 1997) to calibrate a model aimed at in-host modeling of the SARS-COV-2 virus in humans. Unfortunately, the details on DE used were unclear in both papers, and no comparison against other metaheuristics was made. Xavier et al. (2020) used the basic DE for calibration of 11 parameters of the human immunological response to COVID-19 model that is based on five ordinary differential equations. Bhaliya and Shah (2020), de Castro et al. (2020) and Gonzalez-Paz et al. (2020) applied Molegro Virtual Docker package that uses Guided DE variant (Thomsen and Christensen 2006) to dock molecules when searching for inhibiting methods against SARS-COV-2 virus. Similar approach was used by Sheybani et al. (2020), but without any discussion on DE algorithm used. Nowakova et al. (2020) used the classical DE (Storn and Price 1997) for selection of subsets of matrix columns to analyze COVID-19 radiographs; again—no comparison against other metaheuristics was provided. Wu et al. (2020) found that among 5 competitors, the algebraic DE variant (Santucci et al. 2016) is the best method for mask-production real-time scheduling task. Discrete hybridization of PSO and DE has also been compared against three other metaheuristics for goods assignment maximization during COVID-19 pandemic and the risk of infection minimization (Zou et al. 2020); the hybrid approach was praised, but it seems to perform better for the infection minimization criterion than for goods assignment problem.

Applications of PSO to various COVID-19 related tasks, apart from epidemiology and image-based diagnostics, were also numerous. Asghari et al. (2020) were looking for a method for fast SARS-COV-2 presence detection; in their study an, unfortunately unspecified, PSO variant found a rather technical application to minimize the bending loss of the specified waveguide of COVID-19-aimed biosensor. Bhonde et al. (2020) applied, unfortunately also undefined, a binary PSO variant when calibrating random forest algorithm for features selection, aiming at detecting an infection of the coronavirus within host. When developing a blood test for the presence of SARS-COV-2 virus, de Freitas-Barbosa et al. (2021) used the PSO variant proposed by Wang et al. (2007) for feature selection and compared it against Evolutionary search approach (Kim et al. 2000). Authors found equal performance of both methods. Issa and Abd Elaziz (2020) proposed PSO hybridized with Ions Motion (IMO, Javidy et al. 2015) algorithm and compared it against five other metaheuristics, including another version of PSO hybridized with Sine Cosine algorithm (SCA, Issa et al. 2018), for finding the longest common consecutive subsequence in SARS-COV-2 genome by means of Fragmented Local Aligner Technique (Issa et al. 2018). PSO hybridized with IMO and SCA were ranked as the two best approaches. Therib et al. (2020) used the basic PSO variant to calibrate PID controller applied to robotic arm maneuvering that is to be used for remote care of COVID-19 patients. Mustopa et al. (2020) applied PSO to classify the opinions of users on the Indonesian mobile application developed to allow authorities to monitor the spread of SARS-COV-2 in population. Hakimah and Kurniawan (2020) compared the basic PSO without inertia weight and undefined version of GA on calibration of a model aimed at forecasting Rupiah exchange rates against USD during COVID-19 pandemic; authors found PSO to be marginally better than GA. Kang et al. (2020) used the basic version of PSO to fitting two parameters of a simple formula relating the differences between modeled and mobile phone-based computed flow of people in the USA during COVID-19 pandemic. Finally, Fister et al. (2020a) showed a much different application of PSO connected with COVID-19 disease; the authors presented a humanities-related study in which they were searching for relationships between words used in COVID-19 research by means of text mining with the help of PSO-ARTM (Fister et al. 2020b) algorithm.

In some studies authors used PSO to solve various methodological problems and suggest (without empirical examples) that the approach may be useful for research related to COVID-19 pandemic. Among them, Machova et al. (2020) presented an application of PSO to lexicon labeling in order to analyze the positive and negative sentiments and opinions of people on various issues; authors finalize the paper suggesting that the method could be used for analyzing moods of people regarding COVID-19 pandemic. Susanto et al. (2020) discussed how various clustering algorithms, including PSO-based ones, could be used within cloud intelligent systems to improve business management during COVID-19 pandemic.

### Applications of other metaheuristics against COVID-19

Apart from DE and PSO, a number of other metaheuristics were used against COVID-19. Some of them were mentioned previously, as they were compared against DE or PSO variants in various papers (Lobato et al. 2020; Saif et al. 2021; Abdel-Basset et al. 2020c; Abd Elaziz et al. 2020a, b; Wu et al. 2020; Zheng et al. 2020a, b; Al-qaness et al. 2020a, b, 2021a; Ardabili et al. 2020; Sazvar et al. 2020; Zhan et al. 2020; Canayaz 2020; El-Kenawy et al. 2020; Goel et al. 2020; Sahlol et al. 2020; Issa and Abd Elaziz 2020). Various other studies, in which applications of metaheuristics not related to DE or PSO for COVID-19 research are presented, are summarized in Table 4. They mainly aimed at image-based detection of COVID-19 cases, but also SARS-CoV-2 epidemiology (Pinter et al. 2020; Yousefpour et al. 2020), solving vehicle routing problems during COVID-19 pandemics (Chen et al. 2020), and modeling the effects of government actions (Miralles-Pechuan et al. 2020).

Putting DE and PSO aside, the most widely used metaheuristics in COVID-19 research are variants of Genetic Algorithms (Holland 1975) and various bio- or physics-inspired approaches proposed in recent 6–7 years by a group of researchers, which codes are made freely available in various programming languages on https://sayedalimirjalili.com/projects page. Such bio-inspired algorithms are also frequently used as competitors in papers in which DE and PSO are applied against COVID-19. Other algorithms used include Artificial Bee Colony (Karaboga and Basturk 2008) and Gravitational Search Algorithm (Rashedi et al. 2009). From studies which show inter-comparison among various metaheuristics for COVID-19 research, it is very difficult to sum up which kinds of methods are more efficient: DE, PSO, GA, or newly proposed inspiration-guided algorithms. Nonetheless, it seems specific to COVID-19 research that it is mainly performed with either the basic variants of DE, PSO or GA that were proposed in the previous millennium, which codes are available in various platforms or computing libraries, or the recently introduced, inspiration based metaheuristics developed by a group of researchers that take care of making their codes freely available in various programming languages, connected with a single web page (https://sayedalimirjalili.com/projects). Interestingly, algorithms that won various Competitions on Evolutionary Computation, even though their codes are frequently also freely available, are almost never used against COVID-19. This may be due to the fact that codes of competition winners are harder to find, descriptions of algorithms are often published in conference proceedings, not journal papers, and codes are generally available in a single programming language. Although the above discussion may lead to some over-simplification, it seems that for the majority of researchers working against COVID-19 the code availability and name recognition of the method were the prime motivations for the choice of particular metaheuristics. Methods with high name recognition include both old and well established classical algorithms like DE, PSO or GA, as well as new metaheuristics that, due to its naming easily focus reader’s attention (Sorensen 2015; Fausto et al. 2020) and are rapidly cited in journal papers.

## Methodological aspects of differential evolution and particle swarm optimization applications

This section focuses on methodological features of DE and PSO algorithms used in COVID-19 research; the application-oriented discussion was given in Sect. 2. Because in the vast majority of studies numerical single-objective variants of DE and PSO were used, they will be of main interest in this section.

### DE and PSO variants used against COVID-19

In the vast majority of applications against COVID-19 the basic versions of DE (Storn and Price 1997) or PSO (Kennedy and Eberhart 1995) are used—see Tables 1 and 3. PSO is almost always used with inertia weight that was technically added later by Shi and Eberhart (1998), but in many of studies authors refer to 1995 paper. In some SARS-COV-2 related papers the reference to the variant used is cited, in others—the algorithm is briefly described, allowing readers to infer that the basic variant is used even though the source is not clearly stated. Unfortunately, in numerous applications of DE or PSO against COVID-19 neither a reference to specific variant nor its description is provided, hence readers de facto do not know which approach was used (such cases are marked with ? in Tables 1 and 3).

Although in COVID-19-research among DE variants the basic one (Storn and Price 1997) is clearly the most popular, some other single-objective numerical DE variants are also applied. Guided DE (Thomsen and Christensen 2006) is used in some studies (Bhaliya and Shah 2020; de Castro et al. 2020; Gonzalez-Paz et al. 2020; Sheybani et al. 2020), as it has been implemented into Molegro Virtual Docking package that is popular for docking molecules in COVID-19 research. iL-SHADE (Brest et al. 2016) is tested against six other optimizers for x-ray image thresholding, but is ranked the poorest approach. The reason for such a poor performance of iL-SHADE is probably the improper usage of linear population size reduction (it is claimed that all algorithms use 30 individuals, without commenting how it affects iL-SHADE), and very low number of allowed function calls (4500) that prevent iL-SHADE from efficiently adapting its control parameters.

To solve bi-objective problems, two multi-objective DE variants were used against COVID-19, but without much success. MODE (Babu et al. 2005) algorithm was said to be used and compared against an unspecified variants of PSO and GA by Singh et al. (2020b), but the results were not clearly discussed. DECMOSA (Zamuda et al. 2009) was used to solve bi-objective problem of balancing costs and disease spread when allocating resources to hospitals (Zheng et al. 2020b), but was generally outperformed by other algorithms.

For non-numerical problem of scheduling real-time mask production, an Algebraic DE (Santucci et al. 2016) algorithm is used; it is ranked the best when compared against four other optimizers (Wu et al. 2020).

Apart from Kennedy and Eberhart’s (1995) version, just a single PSO variant was used for single-objective numerical COVID-19 related problem. Although some authors (Dutra et al. 2020; Kergassner et al. 2020) refer to PSO reviews or parameters-related studies published in the present century, from the discussion it is clear that they still use the basic PSO variant. The exception is the hierarchical PSO with time varying coefficients (Ratnaweera et al. 2004) that was used by Godio et al. (2020) for calibration of SEIR model.

Non-basic PSO variants were used mainly for feature selection. For this task a binary PSO (Too et al. 2019) was tested in Canayaz (2020). An older version of binary PSO (Kennedy and Eberhart 1997) was compared against 3 other metaheuristics in Too and Mirjalili (2020); all methods achieved very similar results. El-Kenawy et al. (2020) used two-step PSO variant proposed for feature selection (Bello et al. 2007) and a numerical PSO hybridized with Grey Wolf Optimizer (Senel et al. 2019); both methods were compared against ten other metaheuristics and ranked poorly. In the same paper (El Kenawy et al. 2020) PSO was hybridized with guided Whale Optimization Algorithm for classification, and this hybrid turned out the best among five compared metaheuristics. De Freitas Barbosa (2021) used PSO variant proposed for feature selection by Wang et al. (2007), and found its performance to be equal with Evolutionary Search (Kim et al. 2000). Sahlol et al. (2020) used an unspecified variant called BPSO for feature selection; in comparison against 9 other metaheuristics on two data sets BPSO ranked 2nd and 4th. For genome sequence search problem, PSO was hybridized with Ions Motion Optimization and Sine Cosine Algorithm (Issa et al. 2018; Issa and Abd Elaziz 2020), and both hybrids performed better than four other metaheuristics. Finally, in a paper loosely related to COVID-19, a specific variant of PSO for association rule text mining was used by Fister et al. (2020a, b).

It is unclear why, despite so large number of DE (Das et al. 2016; Opara and Arabas 2019) and PSO (Bonyadi and Michalewicz 2017a; Harrison et al. 2018) variants were proposed in recent 2 decades, among which some (e.g. L-SAHDE, Tanabe and Fukunaga 2014) achieved great successes in wide scale competitions among metaheuristics, for numerical problems related with COVID-19 almost solely the basic DE and PSO algorithms were applied. It seems that successful noisy multi-objective variants (Rakshit and Konar 2015) are also ignored. The only explanation seems to be simplicity, popularity and availability of the codes implemented in various languages or computing platforms. The wide-scale development of DE and PSO seems to be missed by the practical users that rapidly, as in the case of early papers on COVID-19, need some optimization tool, but do not work everyday in the field of metaheuristics. Considering the relatively wide application against COVID-19 of various inspiration-guided metaheuristics proposed ad hoc in recent years that are freely available in different computing languages, the problem of public attention and code sharing require re-consideration by the researchers working on PSO and DE development.

### Number of allowed function calls

The maximum number of function evaluations (calls) is a very important factor that may determine both the quality of solutions that are to be found, and the ranking of metaheuristics, if they are to be compared in particular study (Piotrowski et al. 2017; Price et al. 2019). Unfortunately, it is frequently neglected and unspecified in COVID-19 related papers.

When the number of function calls is given explicitly (or may be inferred from other information given in the particular study), two distinct approaches are seen in SARS-COV-2 related papers. In many studies the number of function calls is probably high enough, maybe even excessive, like when 160,000 calls are allowed for solving 10-dimensional problem (Ames et al. 2020), 50,000 for 3-dimensional problem (de Falco et al. 2020), 500,000 for up to 4-dimensional problems (Ardabili et al. 2020), 300,000 for 6-dimensional problem (Oliveira et al. 2021) or 1,000,000 for 5-dimensional problem (Paggi 2020b). Of course, the number of function calls needed to find a global optimum may be high even for some low-dimensional problems (e.g. Price et al. 2019; Yue et al. 2019), but routinely for benchmarking metaheuristics the number of function calls is set lower (e.g. Awad et al. 2016b; Liang et al. 2013) than in the mentioned COVID-19 related papers. In the study by Rica and Ruz (2020) 15,000 function calls is used to find 5 parameters of SIR model, what is relatively low, but probably a sufficient value. As a result, in a number of papers the quality of the solutions found for COVID-19 related problems should not be affected by the computational budget.

Unfortunately, in over 50% of studies in which the number of function calls is specified it is low, ranging from a few hundreds (e.g. Ezzat et al. 2020) to a few thousands (e.g. Comunian et al. 2020). This may be sufficient if the problem is simple enough, but otherwise may affect the quality of the final solution found by the algorithm. It is unfortunate that this may indeed take place in some papers devoted to important problems related with COVID-19 disease.

### Number of repetitions

Evolutionary or Swarm Intelligence Algorithms are stochastic in nature. As a result, in each run a different solution may be found, and many runs are needed to collect a sufficient sample to compare different metaheuristics, or to find out how diverse the quality of solutions found may be. When various algorithms are professionally compared, the number of repetitions is pre-specified, often to a few dozens (e.g. Price et al. 2019; Awad et al. 2016b; Liang et al. 2013). In COVID-19 related papers the number of runs, or repetitions of different algorithms is sometimes unspecified, or may be inferred from the study to be 1. This suggests that the solutions found for the majority of COVID-19 related problems for which DE or PSO were used might be obtained by chance.

However, in some studies the number of runs is provided, and vary between 10 (e.g. Comunian et al. 2020; Sheybani et al. 2020; Bhaliya and Shah 2020; Hakimah and Kurniawan 2020), which is rather low, to 50 or more (Nowakova et al. 2020; Wu et al. 2020; Dutra et al. 2020; Godio et al. 2020). Considering how frequently this issue is ignored, any repetition of numerical experiments support the quality of research. Unfortunately, the statistical comparison of the results is almost never performed. One may only mention here that the problems related with using statistical tests in medicine are under endless debate for many years (Jamart 1992; Strasak et al. 2007; Fernandes-Taylor et al. 2011).

### Population size

Because in the majority of DE and PSO applications against COVID-19 mainly the basic variants are used, the discussion of the choice of control parameters is relatively simple. The population size is the main factor affecting the performance of DE (Mallipeddi and Suganthan 2008; Piotrowski 2017) and PSO (Piotrowski et al. 2020). It is often assumed that it may need to be scaled with the problem dimensionality, or the number of allowed function calls (Price et al. 2019), but in many COVID-19 related applications not all such information is available.

In the majority of DE applications against COVID-19 authors do not clarify the population size used. The impact of the population size on the results is almost never analyzed, with the exception of bi-objective study by Zou et al. (2020) aimed at PSO-DE hybrid, for which 30 individuals turned out the best choice. When population size is given (see Table 1), it almost always ranges from 15 to 50 individuals. The exception is noted in Ames et al. (2020) paper, in which an unspecified DE algorithm with population size set to 400 is used to calibrate 5 and 10 parameters of SIR and SIHRD models, respectively. In such paper the number of function calls allowed is high (160,000). Unfortunately, authors use three different metaheuristics but do not discuss the results obtained by DE variant. On the other hand, in Sainz-Pardo and Valero (2020) only 5 individuals are used to find solutions of a multi-dimensional problem.

The values of population size between 15 and 50 that are often used are rather too small for the classical DE variant; the recommended values are 10 times larger than the problem dimensionality (Storn and Price 1997), or 100 individuals (Piotrowski 2017). However, as DE is generally used to solve low-dimensional problems (with up to 10 dimensions), such small population size may be sufficient as long as the fitness landscape is relatively uncomplicated. Otherwise, small population size used for the basic DE variant would probably result in premature convergence to a local optimum.

Much more diversified population (or swarm) sizes are used for PSO in COVID-19 related papers. Too and Mirjalili (2020) compared binary algorithms, including PSO, with population size set to only 10. Al-quaness et al. (2020a, b), Canayaz (2020), El-Kenawy et al. (2020), de Freitas Barbosa (2021), Goel et al. (2020) and Sahlol et al. (2020) and Abd Elaziz et al. (2020b) used between 15 and 30 particles in their studies. On the other hand, Ardabili et al. (2020), Godio et al. (2020), Kergassner et al. (2020), Paggi (2020a), Fister et al. (2020a) and Dutra et al. (2020) used between 100 and 500 particles, and Paggi (2020b) decided even for 1000 particles. As the problems to be solved by PSO are generally similar in nature and in dimensionality to those addressed by DE algorithms, such diverse choices of PSO population size may be surprising. However, as recently pointed out in Piotrowski et al. (2020), despite classically PSO algorithms are used with 20–50 particles, large number of PSO algorithms including the basic PSO (Eberhart and Kennedy 1995) de facto performs best with much larger swarms, with a few hundreds of particles. This discrepancy between the classical approach, based on experiments performed in the late 1990’s, and observed performance on problems currently widely used in PSO literature may be the reason of so large differences in swarm sizes noted in COVID-19 related papers: some authors follow classical choices, some set higher values as they note that it improves the quality of solutions that are found.

### Other DE and PSO control parameters

Apart from the population size, both DE and PSO have some additional control parameters: scale factor (F) and crossover (CR) in the case of DE, c_1_ and c_2_ acceleration coefficients and *w* inertia weight in the case of PSO. A number of studies were performed to specify the best values of acceleration coefficients (Clerc and Kennedy 2002; Samal et al. 2007; Bonyadi and Michalewicz 2017b; Cleghorn and Engelbrecht 2018) or inertia weights (Shi and Eberhart 1998; Suresh et al. 2008) in PSO; all three parameters are interrelated (Clerc and Kennedy 2002; Eberhart and Shi 2000). In the case of DE, the impact of scale factor (Ronkkonen et al. 2005; Sharma et al. 2019) or crossover (Zaharie 2009; Weber et al. 2013) on the performance has also been analyzed, but in recent DE variants both control parameters are often made adaptive (Ghosh et al. 2011; Das et al. 2016; Al-Dabbagh et al. 2018). Unfortunately, such adaptive new variants were not used against COVID-19 in 2020, with exception of Singh et al. (2020b) and Abdel-Basset et al. (2020c) studies, which however lack sufficient details of DE application. The choice of non-adaptive control parameters may highly impact the quality of the solution found, but this would depend on the specific problem.

Unfortunately, authors frequently do not mention values of control parameters when solving COVID-19 related problems. When they do, in the case of DE algorithms F and CR parameters are often set between 0.5 and 0.9 (de Falco et al. 2020; Lobato et al. 2020; Quaranta et al. 2020; Libotte et al. 2020; Nowakova et al. 2020). The scale factor is frequently (Lobato et al. 2009; Saif et al. 2021; Nowakova et al. 2020) set to 0.9, what agrees with the well-known finding by Ronkkonen et al. (2005) that F should be set between 0.4 and 0.95, with 0.9 being often the best choice. Rica and Ruz (2020) randomly generated F from [0.5,1.0] interval in each generation. However, Singh et al. (2020a) set F to 0.1 for COVID-19 related Convolutional Neural Network’s hyperparameter tuning. Sainz-Pardo and Valero (2020) randomly generated F from [0.0,1.0] interval in each generation, and skipped crossover at all. The choice of CR is more disputable, as it highly depends on the problem—for separable ones the low CR values are needed (i.e. about 0.1), for non-separable—high (i.e. 0.9 or higher, Zaharie et al. 2009). As it is difficult to assume separability of COVID-19-related real world problems, one may expect that higher CR should be used—and indeed researchers frequently choose CR ≈ 0.8–0.9 (de Falco et al. 2020; Lobato et al. 2020; Libotte et al. 2020; Nowakova et al. 2020). However, Saif et al. (2021) used CR = 0.2 as for separable problems, and Singh et al. (2020a) decided for 0.5. It may be concluded that, although the control parameters of DE algorithms are not made adaptive, their choices (if provided) are generally justified by the findings from DE-oriented literature.

Authors of COVID-19 related papers that use PSO often choose c_1_ = c_2_ = 2 (Al-quaness et al. 2020a, b; Dutra et al. 2020; He et al. 2020b; Van Tinh 2020a, b; Canayaz 2020; El-Kenawy et al. 2020; Abd Elaziz et al. 2020b; Fister et al. 2020a; Too and Mirjalili 2020)—a setting that was initially suggested by Eberhart and Kennedy (1995) and is also re-supported by some reviews (Marini and Walczak 2015). Another popular choice in papers aimed at COVID-19 pandemic is c_1_ = c_2_ = 0.5 (Paggi 2020a, b; Bhonde et al. 2020; Issa and Abd-Elaziz 2020), which is hard to explain based on the classical PSO-related literature. Just once, in Kergassner et al. (2020) the setting c_1_ = c_2_ = 1.49445 suggested by Clerc and Kennedy (2002) and Eberhart and Shi (2000) is “almost” used (almost, as authors technically chosen c_1_ = c_2_ = 1.4696172). This choice needs to be coupled with *w* = 0.729. Some authors used other c_1_ and c_2_ settings (e.g. in Zreiq et al. 2020, c_1_ = c_2_ = 0.75), unfortunately without justification. Very rarely in COVID-19 related papers c_1_ ≠ c_2_ (in Oliveira et al. 2021 and Jorge et al. 2020, c_1_ = 0.1, c_2_ = 0.3), and the reason for unequal setting of both coefficients is not discussed. Inertia weights are frequently made decreasing during search (Al-quaness et al. 2020a, b; Paggi 2020a, b; Van Tinh 2020a, b; Canayaz 2020; El-Kenawy et al. 2020; Abd Elaziz et al. 2020b; Too and Mirjalili 2020), as suggested in Shi and Eberhart (1998). However, the fixed inertia weight set to 0.9 (Bhonde et al. 2020; Dutra et al. 2020), 0.7 (Fister et al. 2020a), or to the value of 0.729 (Kergassner et al. 2020) suggested in Clerc and Kennedy (2002) (which should be accompanied by the specific setting of acceleration coefficients), and an unexpected very small value of 0.2 (Issa and Abd Elaziz 2020) are also used. In PSO-DE bi-objective hybrid (Zou et al. 2020) extremely low values of acceleration coefficients and inertia weight were used, but this may be due to the hybridization interactions with DE counterpart. Hence, as in the case of DE, one may conclude that in the majority of studies that use PSO against COVID-19 in which inertia weight and acceleration coefficients are specified, their choices follow suggestions from the PSO literature. However, in some papers control parameters seems to be too small (Oliveira et al. 2021; Jorge et al. 2020; Issa and Abd Elaziz 2020), what could lead to the premature convergence.

### Comparison of performance

Choosing the better method among the competitors is very important for practical users, even though various approaches to the problem of comparison between metaheuristics are still debated in the literature (Garcia and Herrera 2008; Crepinsek et al. 2016; Hussain et al. 2019; Halim et al. 2021). In the majority of papers in which DE or PSO are used to solve COVID-19 related problems, only one variant of a single optimization method is used. Hence, no comparison of performance between various methods can be done, and the quality of the results obtained cannot be validated. Nonetheless, in some COVID-19 related studies two or more metaheuristics are compared. It is difficult to generalize the results, as each study address a different optimization problem, or use different data sets. In many studies either the basic DE, basic PSO or both these algorithms are used, but in each paper they are compared against much different other metaheuristics. In some studies it is reported that various metaheuristics are used, but finally their results are not given (Ames et al. 2020; Singh et al. 2020b; Naraigh and Byrne 2020; Zhan et al. 2020).

It is impossible to claim whether DE or PSO overall perform better against COVID-19. In Saif et al. (2021) study that aimed at calibration of ANFIS parameters PSO clearly outperforms DE; PSO ranks 2–3nd out of eight compared algorithms, DE is among two the worst methods. However, this may be due to the low computational budget (only 5000 function calls are allowed) which favor PSO (Piotrowski et al. 2017), or low population size, set to 25 for all algorithms (what is inappropriate for DE). Hagshenas et al. (2020) found PSO marginally better than DE for Multilayer Perceptron ANN calibration when studying the impact of environmental factors on COVID-19 pandemic; but again both the number of function calls and the population size were very small, favoring PSO. Zheng et al. (2020a) found DE variant slightly better than CLPSO (Liang et al. 2006) for resources allocation problem, but the details on such important features like computational time or population size were unspecified.

When DE or PSO are compared against other metaheuristics, but not against each other, DE performs either very well (Abd Elaziz et al. 2020a; Wu et al. 2020), or poorly (in the discussed earlier case of iL-SHADE, Abdel-Basset et al. 2020c). When DE is not considered, PSO perform very well against other metaheuristics only in Issa and Abd Elaziz (2020), it more frequently ranks moderately (Al-quaness et al. 2020a, b; Ardabili et al. 2020; Canayaz 2020; Sahlol et al. 2020; Abd Elaziz et al. 2020b; Too and Mirjalili 2020) or poorly (Sazvar et al. 2020; Zhan et al. 2020; El-Kenawy et al. 2020; Goel et al. 2020; Al-qaness et al. 2021a). Based on the above summary, it is impossible to give a hint whether PSO or DE is better suited for solving COVID-19 related cases; the results seems also to not necessarily be clear for a specific kind of problems.

The problem with the contradictory findings regarding the superiority of some methods over the others that comes up when reading different papers related to COVID-19 is rather an effect of the way the comparison is organized. To some extent it may be due to the low numbers of allowed function calls and low population sizes used in vast majority of COVID-19 related papers in which various metaheuristics are compared. The reader is referred to Table 5 for a summary of both factors in papers in which a comparison between various metaheuristics is shown. With a very few exceptions, the maximum number of function calls is not higher than 5.000, and the population size is set between 10 and 30. Such low numbers of allowed function calls and population size prefers variants of algorithms that converge quickly over those with enhanced exploration capabilities, and make the whole comparison more prone to the manual choice of control parameters, or even to the random effects.

**Table 5:** Summary of the number of function calls and the population size used in metaheuristics applied to solve COVID-19 related problems. Only papers in which the comparison between various metaheuristics is performed are shown. For details on each paper, please see Tables 1, 2, 3 and 4

Paper	Number of function calls	Population size
Lobato et al. (2020)	6.250	25
Saif et al. (2021)	5.000	25
Abdel_Basset et al. (2020c)	4.500	30
Abd Elaziz et al. (2020a)	?	?
Punitha et al. (2020)	?	?
Haghshenas et al. (2020)	?	?
Wu et al. (2020)	100.000	?
Zheng et al. (2020a)	?	?
Zheng et al. (2020b	?	?
Zou et al. (2020)	Other	30 (PSO-DE) unclear for others
Al-quaness et al. (2020a )	2.500	25
Al-quaness et al. (2020b)	2.500	25
Al-quaness et al. (2021)	?	?
Sazvar et al. (2020)	?	?
Too and Mirjalili (2020)	1.000	10
Canayaz (2020)	2.000	20
El-Kenawy et al. (2020)	400–800	10–20
Goel et al. (2020)	900	30
Sahlol et al. (2020)	300	15
Abd Elaziz et al. (2020b)	2.000	20
Issa and Abd-Elaziz (2020)	?	40–700
Alrashidi et al. (2020)	?	?
Abdel-Basset et al. (2020b)	3.000	20
Chen et al. (2020)	?	?
Lu et al. (2021)	?	?
Al-qaness et al. (2021b)	3.000	30
Yousri et al. (2021)	750	15

## Conclusions

In scientific papers related to COVID-19 pandemic both DE and PSO algorithms found numerous applications. They are most widely used for calibration of epidemiology models and for optimization of parameters or selection of features for image-based diagnostics. However, both DE and PSO are also applied to COVID-19-related studies in much different fields of science, from management to linguistics. In the majority of papers DE and PSO variants are compared neither against each other, nor against any other metaheuristics. From studies in which such comparison is performed, no clear picture of superiority of one method against the other emerges.

Despite the rapid development of DE and PSO algorithms in recent two decades, in studies addressing COVID-19 related problems mainly the basic DE (Storn and Price 1997) or the basic PSO (Eberhart and Kennedy 1995) variants are used. Apart from PSO or DE versions that were developed for feature selection problems, the newer variants of both methods are ignored in COVID-19 research. It may be surprising, because the recent variants show much better performance than their classical versions in numerous papers, and the codes of various successful versions are widely available from different authors and web pages of Competitions on Evolutionary Computation that are held regularly every year.

In the majority of studies related to COVID-19 disease that use DE or PSO algorithms, one may note the lack of information on such important methodological details like dimensionality of the problem that is being optimized, the number of repetitions (runs) made, the number of function calls allowed, or the choice of control parameter settings. In those studies where particular details are reported, some choices made are frequently inadequate, and highly differ for each study. The allowed computational budget was set very low in many papers, especially those in which various metaheuristics were compared against each other, but excessively high in some others. As mainly the basic, non-adaptive variants of DE and PSO were used, the setting of their control parameters was especially important. With a few exceptions, the population size was often set small and fixed for all algorithms used (if there were more than one), what favors PSO over DE methods, as the latter often require higher population. However, contrary to the population size, the values of crossover and mutation factor in DE, as well as acceleration coefficients and inertia weight in PSO are often appropriate and based on the literature.

Researchers working on COVID-19 pandemic often seek for simple and easily available optimization methods, either new or those highly cited. It seems that the availability of codes in various computing languages and either the novelty, or the name recognition and the number of citations are the primary reasons for choosing particular algorithm. Neither good performance in Competitions on Evolutionary Computation nor wide-scale theoretical or empirical discussion in specialized literature seems to be of any importance for practitioners. Hence, the majority of researchers working on problems related with COVID-19 disease use either the basic variants of DE (Storn and Price 1997) or PSO (Eberhart and Kennedy 1995; Shi and Eberhart 1998) that are widely cited and easily available in variety of computing platforms, or those inspiration-guided metaheuristics that were proposed very recently, have appealing names, and which codes are easily and freely available.

## References

[CR1] Abbas HA (2002). An evolutionary artificial neural networks approach for brest cancer diagnosis. Artif Intell Med.

[CR2] Abd Elaziz M, Ewees MAA, Hassanien AE (2017). Whale optimization algorithm and moth-flame optimization for multilevel thresholding image segmentation. Exp Syst Appl.

[CR3] Abd Elaziz M, Hosny KH, Salah A, Darwish MM, Lu S, Sahlol AT (2020). New machine learning method for image based diagnosis of COVID-19. PLoS ONE.

[CR4] Abd Elaziz M, Ewees AA, Yousri D, Arwelfali HSN, Awad QA, Lu S, Al-Qaness MAA (2020). An improved marine predators algorithm with fuzzy entropy for multi-level thresholding: real world example of COVID-19 CT image segmentation. IEEE Access.

[CR5] Abdel-Basset M, Zhou Y, Ismail M (2018). An improved cuckoo search algorithm for integer programming problems. Int J Comput Sci Math.

[CR6] Abdel-Basset M, Chang V, Mohamed R (2020). A novel equilibrium optimization algorithm for multi-thresholding image segmentation problems. Neural Comput Appl.

[CR7] Abdel-Basset M, Mohamed R, Elhoseny M, Chakraborty RK, Ryan M (2020). A hybrid COVID-19 detection model using an improved marine predators algorithm and a ranking-based diversity reduction strategy. IEEE Access.

[CR8] Abdel-Basset M, Chang V, Mohamed R (2020). HSMA-WOA: a hybrid novel Slime mould algorithm with whale optimization algorithm for tackling the image segmentation problem of chest X-ray images. Appl Soft Comput.

[CR9] Abuin P, Anderson A, Ferramosca A, Hernandez-Vargas AE, Gonzalez AH (2020). Characterization of SARS-CoV-2 dynamics in the hose. Annu Rev Control.

[CR10] Ahmad A, Garhawal S, Ray SK, Kumar G, Malebary SJ, Barukab OM (2020). The number of confirmed cases of covid-19 by using machine learning: methods and challenges. Arch Comput Methods Eng.

[CR11] Ahuja RK, Orlin JB, Tiwari A (2000). A greedy genetic algorithm for the quadratic assignment problem. Comput Oper Res.

[CR12] Albahri OS, Zaidan AA, Albahri AS, Zaidan BB, Abdulkareem KH, Al-qaysi ZT, Alamoodi AH, Aleesa AM, Chyad MA, Alesa RM, Kem LC, Lakulu MM, Ibrahim AB, Rashid NA (2020). Systematic review of artificial intelligence techniques in the detection and classification of COVID-19 medical images in terms of evaluation and benchmarking: taxonomy analysis, challenges, future solutions and methodological aspects. J Infect Public Health.

[CR13] Al-Betar MA, Abdi Z, Alyasseri A, Awadallah MA, Abu Doush I (2020). Coronavirus herd immunity optimizer (CHIO). Neural Comput Appl.

[CR14] Al-Dabbagh RD, Neri F, Idris N, Baba MS (2018). Algorithmic design issues in adaptive differential evolution schemes: review and taxonomy. Swarm Evol Comput.

[CR15] Al-Hussein ABA, Tahir FR (2020) Epidemiological characteristics of COVID-19 ongoing epidemic in Iraq. Bull World Health Organ (Preprint), e-pub. 10.2471/BLT.20.257907

[CR16] Al-Madi N, Faris H, Mirjalili S (2019). Binary multi-verse optimization algorithm for global optimization and discrete problems. Int J Mach Learn Cybern.

[CR17] Al Moubayed N, Petrovski A, McCall J (2014). D2MOPSO: MOPSO based on decomposition and dominance with archiving using crowding distance in objective and solution spaces. Evol Comput.

[CR18] Al-qaness MA, Ewees AA, Fan H, Abualigah L, Abd Elaziz M (2020). Marine predators algorithm for forecasting confirmed cases of COVID-19 in Italy, USA, Iran and Korea. Int J Environ Res Public Health.

[CR19] Al-qaness MA, Ewees AA, Fan H, Abd Elaziz M (2020). Optimization method for forecasting confirmed cases of COVID-19 in China. J Clin Med.

[CR20] Al-qaness MA, Saba AI, Elsheikh AE, Abd Elaziz M, Ibrahim RA, Lu S, Hemedan AA, Shanmugan S, Ewees AE (2021). Efficient artificial intelligence forecasting models for COVID-19 outbreak in Russia and Brazil. Process Saf Environ Prot.

[CR21] Al-qaness MAA, Fan H, Ewees AA, Yousri D, Elaziz MA (2021). Improved ANFIS model for forecasting Wuhan city air quality and analysis COVID-19 lockdown impacts on air quality. Environ Res.

[CR22] Alrashidi M (2020). Social distancing in indoor spaces: AN intelligent guide based on the Internet of Things: COVID-19 as a case study. Computers.

[CR23] Altan A, Karasu S (2020). Recognition of COVID-19 disease from X-ray images by hybrid model consisting of 2D curvelet transform, chaotic salp swarm algorithm and deep learning technique. Chaos Solitons Fractals.

[CR24] Al-Tashi Q, Abdul Kadir SJ, Rais HM, Mirjalili S, Alhussian H (2019). Binary optimization using hybrid grey wolf optimization for feature selection. IEEE Access.

[CR25] Ames AD, Molnar TG, Singletary AW, Orosz G (2020) Safety-critical control of active interventions for COVID-19 mitigation. medRxiv preprint. 10.1101/2020.06.17.20133264 10.1109/ACCESS.2020.3029558 PMC8545284 34812361

[CR26] Anand N, Sabarinath A, Geetha S, Somanath S (2020). Predicting the spread of COVID-19 using SIR model augmented to incorporate quarantine and testing. Trans Indian Natl Acad Eng.

[CR27] Ardabili SF, Mosavi A, Ghamisi P, Ferdynand F, Varkonyi-Koczy AR, Reuter U, Rabczuk T, Atkinson PM (2020). COVID-19 outbreak prediction with machine learning. Algorithms.

[CR28] Arora N, Banerjee AK, Narasu ML (2020). The role of artificial intelligence in tackling COVID-19. Future Virol.

[CR29] Asghar MA, Razzaq S, Rasheed S, Fawad (2020) A robust technique for detecting SARS-COV-2 from x-ray image using 2D convolutional neural network and particle swarm optimization. In: 14th International conference on open source systems and technologies (ICOSST). 10.1109/ICOSST51357.2020.9333084

[CR30] Asghari A, Wang C, Yoo KM, Dalir H, Chen RT (2020) Fast accurate point of care COVID-19 pandemic diagnosis enabled through advanced lab-on-a-chip optical biosensors: opportunities and challenges. arXiv:2008.08572 10.1063/5.0022211 PMC8427516 34552683

[CR31] Atashpaz-Gargari E, Lucas C (2007) Imperialist competitive algorithm: an algorithm for optimization inspired by imperialistic competition. In: IEEE congress on evolutionary computation, Singapore. Piscataway, IEEE

[CR32] Awad NH, Ali MZ, Suganthan PN, Reynolds RG (2016a) An ensemble sinusoidal parameter adaptation incorporated with L-SHADE for solving CEC2014 benchmark problems. In: Proceedings of the IEEE congress on evolutionary computation, Vancouver, Canada. 10.1109/CEC.2016.7744163

[CR33] Awad NH, Ali MZ, Liang JJ, Qu BY, Suganthan PN (2016b) Problem definitions and evaluation criteria for the CEC 2017 special session and competition on single objective bound constrained real-parameter numerical optimization. Technical Report, Nanyang Technological University, Singapore

[CR34] Babu BV, Chakole PG, Mubeen KHS (2005). Multiobjective differential evolution (MODE) for optimization of adiabatic styrene reactor. Chem Eng Sci.

[CR35] Babukarthik RG, Adiga VAK, Sambasivam G, Chandramohan D, Amudhavel J (2020). Prediction of COVID-19 using genetic deep learning convolutional neural network (GDCNN). IEEE Access.

[CR36] Bao X, Jia H, Lang C (2019). A novel hybrid Harris hawks optimization for color image multilevel thresholding segmentation. IEEE Access.

[CR37] Baraldi P, Bonfranti G, Zio E (2018). Differential evolution-based multi-objective optimization for the definition of a health indicator for fault diagnostics and prognostics. Mech Syst Signal Process.

[CR38] Bello R, Gomez Y, Nowe A, Garcia MM (2007) Two-step particle swarm optimization to solve the feature selection problem. In: Seventh international conference on intelligent systems design and applications (ISDA 2007), pp 691–696

[CR39] Bertuzzo E, Mari L, Pasetto D, Miccoli S, Casagrandi R, Gatto M, Rinaldo A (2020). The geography of COVID-19 spread in Italy and implications for the relaxation of confinement measures. Nat Commun.

[CR40] Bhaliya J, Shah V (2020). Identification of potent COVID-19 main protease (Mpro) inhibitors from Curcumin analogues by molecular docking analysis. Int J Adv Res Ideas and Innovations in Technology.

[CR41] Bhonde SB, Prasad JR, Bhati M (2020). Predictive analytics to combat with COVID-19 using genome sequencing. SSRN.

[CR42] Bonyadi MR, Michalewicz Z (2017). Particle swarm optimization for single objective continuous space problems: a review. Evol Comput.

[CR43] Bonyadi MR, Michalewicz Z (2017). Impacts of coefficients on movement patterns in the particle swarm optimization algorithm. IEEE Trans Evol Comput.

[CR44] Boubaker S (2017). Identification of nonlinear Hammerstein system using mixed integer-real coded particle swarm optimization: application to the electric daily peak-load forecasting. Nonlinear Dyn.

[CR45] Bouchekara H (2017). Most valuable player algorithm: a novel optimization algorithm inspired from sport. Oper Res Int J.

[CR46] Bowman VE, Silk DS, Darlymple U, Woods DC (2020) Uncertainty quantification for epidemiological forecasts of COVID-19 through combinations of model predictions. arXiv:2006.10714v2 10.1177/09622802221109523 PMC9272045 35799481

[CR47] Brest J, Greiner S, Boskovic B, Mernik M, Zumer V (2006). Self-adapting control parameters in differential evolution: a comparative study on numerical benchmark problems. IEEE Trans Evol Comput.

[CR48] Brest J, Maučec MS, Bošković B (2016) IL-SHADE: improved L-SHADE algorithm for single objective real-parameter optimization. In: 2016 IEEE congress on evolutionary computation, CEC, IEEE

[CR49] Brest J, Maučec MS, Bŏsković B (2019) The 100-digit challenge: algorithm jDE100. In: Proceedings of the 2019 IEEE congress on evolutionary computation, Wellington, New Zealand, pp 19–26

[CR50] Bullock J, Luccioni A, Pham KH, Lam CSN, Luengo-Oroz M (2020) Mapping the landscape of artificial intelligence applications against COVID-19. arXiv:2003.11336v2

[CR51] Canayaz M (2020). MH-COVIDNet: diagnosis of COVID-19 using deep neural networks and meta-heuristic-based feature selection on X-ray images. Biomed Signal Process Control.

[CR52] Cantun-Avila KB, Gonzalez-Sanchez D, Diaz-Infante S, Penunuri F (2021). Optimizing functionals using differential evolution. Eng Appl Artif Intell.

[CR53] Carrasco J, Garcia S, Rueda MM, Das S, Herrera F (2020). Recent trends in the use of statistical tests for comparing swarm and evolutionary computing algorithms: practical guidelines and a critical review. Swarm Evolut Comput.

[CR54] Casciati S (2008). Stiffness identification and damage localization via differential evolution algorithms. Struct Control Health Monit.

[CR55] Chen D, Pan S, Chen Q, Liu J (2020). Vehicle routing problem of contactless joint distribution service during COVID-19 pandemic. Transp Res Interdiscip Perspect.

[CR56] Cheng S, Lu H, Lai XJ, Shi YH (2018). A quarter century of particle swarm optimization. Complex Intell Syst.

[CR57] Cheng C, Barcelo J, Hartnett AS, Kubinec R, Messerschmidt L (2020). COVID-19 government response event dataset (CoronaNet vol 1.0). Nat Hum Behav.

[CR58] Cheng T, Fan T, Wang L (2020b) Genetic constrained graph variational autoencoder (GCGVAE) for COVID-19 drug discovery. preprint raw.githubuscerontent.com.

[CR59] Chiroma H, Ezugwu AE, Jauro F, Al-Garadi MA, Abdullahi IN, Shuib L (2020) Early survey with bibliometric analysis on machine learning approaches in controlling coronavirus. medRxiv preprint 10.1101/2020.11.04.20225698 10.7717/peerj-cs.313 PMC7924648 33816964

[CR60] Cholissodin I, Sutrisno S, Santoso N, Soebroto AA, Hindayat N, Rochman NT (2020). Smart development of Big Data App for determining the modeling of COVID-19 medical compounds using deep AI core engine system. J Phys Conf Ser.

[CR61] Cleghorn CW, Engelbrecht AP (2018). Particle swarm stability: a theoretical extension using the non-stagnate distribution assumption. Swarm Intell.

[CR62] Clerc M, Kennedy J (2002). The particle swarm—explosion, stability, and convergence in a multidimensional complex space. IEEE Trans Evol Comput.

[CR63] Comunian A, Gaburro R, Giudici M (2020). Inversion of a SIR-based model: a critical analysis about the application to COVID-19 epidemic. Physica D.

[CR64] Corbacho Abelaira MD, Corbacho Abelaire F, Ruano-Ravina A, Fernandez-Villar A (2021). Use of conventional chest imaging and artificial intelligence in COVID-19 infection. A review of the literature. Open Respir Arch.

[CR65] Cordelli E, Tortora M, Sicilia R, Soda P (2020) Time-window SIQR analysis of COVID-19 outbreak and containment measures in Italy. In: IEEE 33rd international symposium on computer-based medical systems (CBMS). 10.1109/CBMS49503.2020.00059

[CR66] Crepinsek M, Liu SH, Mernik M (2013). Exploration and exploitation in evolutionary algorithms: a survey. ACM Comput Surv.

[CR67] Crepinsek M, Liu SH, Mernik L, Mernik M (2016). Is a comparison of results meaningful from the inexact replications of computational experiments?. Soft Comput.

[CR68] Das S, Abraham A, Konar A (2006) Spatial information based image segmentation using a modified particle swarm optimization algorithm. In: Sixth international conference on intelligent systems design and applications, Jinan, China, IEEE, pp 438–444

[CR69] Das S, Abrajam A, Konar A (2008). Particle swarm optimization and differential evolution algorithms: technical analysis, applicationa and hybridization perspectives. Stud Comput Intell (SCI).

[CR70] Das S, Mullick SS, Suganthan PN (2016). Recent advances in differential evolution—an updated survey. Swarm Evol Comput.

[CR71] Davies NG, Klepac P, Liu Y, Prem K, Jit M, CMMID COVID-19 working group, Eggo RM (2020) Age-dependent effects in the transmission and control of COVID-19 epidemics. Nat Med 26:1205–1211 10.1038/s41591-020-0962-9 32546824

[CR72] Deb K, Pratap A, Agarwal S, Meyarivan T (2002). A fast and elitist multiobjective genetic algorithm: NSGA-II. IEEE Trans Evol Comput.

[CR73] de Camino Beck T (2020) A modified SEIR model with confinement and lockdown of COVID-19 for Costa Rica. medRxiv preprint, 10.1101/2020.05.19.20106492

[CR74] de Castro AA, Assis LC, Ramalho TC, La Porta FA (2020) New in silico insights into the application of the (hydroxy)chloroquine with macrolide antibiotics cocrystals against the SARS-CoV-2 virus. researchsquare.com 10.21203/rs.3.rs-66640/v1

[CR75] de Falco I, Della Cioppa A, Scafuri U, Tarantino E (2020) Coronavirus Covid–19 spreading in Italy: optimizing an epidemiological model with dynamic social distancing through differential evolution. arXiv:2004.00553v3

[CR76] de Freitas Barbosa VA, Carneiro Gomes J, de Santana MA, de Almeida Albuquerque JE, de Souza RG, de Souza RE, dos Santos WP (2021) Heg.IA: an intelligent system to support diagnosis of Covid-19 based on blood tests. Research on Biomedical Engineering. 10.1007/s42600-020-00112-5

[CR77] Derrac J, Garcia S, Hui S, Suganthan PN, Herrera F (2014). Analyzing convergence performance of evolutionary algorithms: a statistical approach. Inf Sci.

[CR78] Du YC, Zhang MX, Cai CY, Zheng YJ (2018) Enhanced biogeography-based optimization for flow-shop scheduling. In: Qiao J, Zhao X, Pan L, Zuo X, Zhang X, Zhang Q, Huang S (eds) Bio-inspired computing: theories and applications. In: Commun. Comput. Inf. Sci., Springer, Singapore, pp 295–306

[CR79] Dutra JCS, da Silva WB, da Costa JMJ (2020) Monitoring and forecasting the number of reported and unreported cases of the COVID-19 epidemic in Brazil using Particle Filter. medRxiv preprint 10.1101/2020.05.27.20115212

[CR80] Eberhart RC, Kennedy J (1995) A new optimizer using particle swarm theory. In: Proceedings of the 6th international symposium on micromachine human science, Nagoya, Japan. IEEE, Piscataway, NJ, USA, pp 39–43

[CR81] Eberhart RC, Shi Y (2000) Comparing inertia weights and constriction factors in particle swarm optimization. In: Proceedings of the 2000 congress on evolutionary computation, La Jolla, CA, USA, IEEE

[CR82] Eiben AE, Hinterding R, Michalewicz Z (1999). Parameter control in evolutionary algorithms. IEEE Trans Evol Comput.

[CR83] Elghamrawy S, Hassanien AE (2020) Diagnosis and prediction model for COVID-19 patient’s response to treatment based on convolutional neural networks and whale optimization algorithm using CT images. medRxiv preprint 10.1101/2020.04.16.20063990

[CR84] El-Kenawy ESM, Ibrahim A, Mirjalili S, Eid MM, Hussein SE (2020). Novel feature selection and voting classifier algorithms for COVID-19 classification in CT images. IEEE Access.

[CR85] Erdmann H, Wachs-Lopes C, Gallao C, Rioberto MP, Rodrigues PS (2015) A study of a firefly meta-heuristics for multithreshold image segmentation. In: Developments in medical image processing and computational vision, Springer, pp 279–295

[CR86] Estrada E (2020). COVID-19 and SARS-CoV-2. Modeling the present, looking at the future. Phys Rep.

[CR87] Ezzat D, Hassanien AE, Ella HA (2020). An optimized deep learning architecture for the diagnosis of COVID-19 disease based on gravitational search optimization. Appl Soft Comput.

[CR88] Fanelli D, Piazza F (2020). Analysis and forecast of COVID-19 spreading in China Italy and France. Chaos Solitons Fractals.

[CR89] Faramarzi A, Heidarinejad M, Mirjalili S, Gandomi AH (2020). Marine predators algorithm: a nature-inspired metaheuristic. Exp Syst Appl.

[CR90] Farhat H, Sakr GE, Kilany R (2020). Deep learning applications in pulmonary medical imaging: recent updates and insights on COVID-19. Mach vis Appl.

[CR91] Fausto F, Reyna-Orta A, Cuevas E, Andrade AG, Perez-Cisneroz M (2020). From ants to whales: metaheuristics for all tastes. Artif Intell Rev.

[CR92] Fernandes N (2020). Economic effects of coronavirus outbreak (COVID-19) on the world economy. SSRN.

[CR93] Fernandes-Taylor S, Hyun JK, Reeder RN, Harris AHS (2011). Common statistical and research design problems in manuscripts submitted to high-impact medical journals. BMC Res Notes.

[CR94] Fetzer T, Hensel L, Hermle J, Roth C (2020). Coronavirus perceptions and economic anxiety. Rev Econ Stat.

[CR95] Fister I Jr, Yang XS, Fister I, Brest J (2012) Memetic firefly algorithm for combinatorial optimization. arXiv:1204.5165

[CR96] Fister I Jr, Fister K, Fister I (2020a) Discovering associations in COVID-19 related research papers. arXiv:2004.03397v1

[CR97] Fister I Jr, Deb S, Fister I (2020b) Population-based metaheuristics for association rule text mining. arXiv:2001.06517v1

[CR98] Forster PM, Forster HI, Evans MJ, Gidden MJ, Jones CD, Keller CA, Lamboll RD, Le Quere C, Rogelj J, Rosen D, Schleussner CF, Richardson TB, Smith CJ, Turnock ST (2020). Current and future global climate impacts resulting from COVID-19. Nat Clim Change.

[CR99] Freitas Reis R, de Melo QB, de Oliveira CJ, Moreira Gomes J, Martins Rocha B, Lobosco M, Weber dos Santos R (2020). Characterization of the COVID-19 pandemic and the impact of uncertainties, mitigation strategies, and underreporting of cases in South Korea, Italy, and Brazil. Chaos Solitons Fractals.

[CR100] Garcia S, Herrera F (2008). An extension on “statistical comparisons of classifiers over multiple data sets” for all pairwise comparisons. J Mach Learn Res.

[CR101] Gatto M, Bertuzzo E, Mari L, Miccoli S, Carraro L, Casagrandia R, Rinaldo A (2020). Spread and dynamics of the COVID-19 epidemic in Italy: effects of emergency containment measures. Proc Natl Acad Sci USA.

[CR102] Ghosh A, Das S, Chowdhury A, Giri R (2011). An improved differential evolution algorithm with fitness-based adaptation of the control parameters. Inf Sci.

[CR103] Gillingham KT, Knittel CR, Li J, Ovaere M, Reduant M (2020). The short-run and long-run effects of COVID-19 on energy and the environment. Joule.

[CR104] Giudici M, Comunian A, Gaburro R (2020) Inversion of a SIR-based model: a critical analysis about the application to COVID-19 epidemic. arXiv:2004.07738v2 10.1016/j.physd.2020.132674 PMC7419377 32834252

[CR105] Glover F (1986). Future paths for integer programming and links to artificial intelligence. Comput Oper Res.

[CR106] Godio A, Pace F, Vergnano A (2020). SEIR modeling of the Italian epidemic of SARS-CoV-2 using computational swarm Intelligence. Int J Environ Res Public Health.

[CR107] Godreev D, Singer P, Michailidis M, Muller M, Ambati SS (2020) Backtesting the predictability of COVID-19. arXiv:2007.11411v1

[CR108] Goel T, Murugan R, Mirjalili S, Chakrabartty DK (2020). OptCoNet: an optimized convolutional neural network for an automatic diagnosis of COVID-19. Appl Intell.

[CR109] Gonzalez-Paz LA, Lossada CA, Mancayo LS, Romero F, Paz JL, Vera-Villalobos J, Perez AE, San-Blas E, Alvarado YJ (2020) Theoretical molecular docking study of the structural disruption of the viral 3CL-protease of COVID-19 induced by binding of capsacin, piperine and curcumin Part 1: a comparative study with chloroquine and hydrochloroquine two antimalaric drugs. ResearchSquare. 10.21203/rs.3.rs-21206/v1

[CR110] Goodfellow I, Bengio Y, Courville A (2016). Deep learning.

[CR111] Hadi MA, Ali HI (2021). Control of COVID-19 system using a novel nonlinear robust control algorithm. Biomed Signal Process Control.

[CR112] Haghani M, Bliemer MCJ, Goerlandt F, Li J (2020). The scientific literature on Coronaviruses, COVID-19 and its associated safety-related research dimensions: A scientometric analysis and scoping review. Saf Sci.

[CR113] Haghshenas SS, Pirouz B, Piro P, Na KS, Cho SE, Geem ZW (2020). Prioritizing and analyzing the role of climate and urban parameters in the confirmed cases of COVID-19 based on artificial intelligence applications. Int J Environ Res Public Health.

[CR114] Hakimah M, Kurniawan M (2020) Integration of double exponential smoothing damped trend with metaheuristic methods to optimize forecasting Rupiah exchange rates against USD during COVID-19 pandemic. Jurnal Ilmu Komputer dan Inform. 10.23917/khif.v6i2.9887

[CR115] Halim AH, Ismail I, Das S (2021). Performance assessment of the metaheuristic optimization algorithms: an exhaustive review. Artif Intell Rev.

[CR116] Harmon SA, Sanford TH, Xu S, Turkbey EB, Roth H, Xu Z, Yang D, Myronenko A, Anderson V, Amalou A, Blain M, Kassin M, Long D, Varble N, Walker SM, Bagci U, Ierardi AM, Stellato E, Plensich GG, Franceschelli G, Girlando C, Irmici G, Labella D, Hammoud D, Malayeri A, Jones E, Summers RM, Choyke PL, Xu D, Flores M, Tamura K, Obinata H, Mori H, Patella F, Cariati M, Carafiello G, An P, Wood BJ, Turkbey B (2020). Artificial intelligence for the detection of COVID-19 pneumonia on chest CT using multinational datasets. Nat Commun.

[CR117] Harrison KR, Engelbrecht AP, Ombuki-Berman BM (2018). Self-adaptive particle swarm optimization: a review and analysis of convergence. Swarm Intell.

[CR118] Hashim FA, Houssein EH, Mabrouk MS, Al-Atabany W, Mirjalili S (2019). Henry gas solubility optimization: a novel physics based algorithm. Futur Gener Comput Syst.

[CR119] He J, Chen G, Jiang Y, Jin R, Shortridge A, Agusti S, He M, Wu J, Duarte CM, Christakos G (2020). Comparative infection modeling and control of COVID-19 transmission patterns in China, South Korea Italy and Iran. Sci Total Environ.

[CR120] He S, Peng Y, Sun K (2020). SEIR modeling of the COVID-19 and its dynamics. Nonlinear Dyn.

[CR121] Heidari AA, Mirjalili S, Faris H, Aljarah I, Mafarja M, Chen H (2019). Harris hawks optimization: algorithm and applications. Futur Gener Comput Syst.

[CR122] Hernandez-Vargas EA, Wilk E, Canini L, Toapanta FR, Binder SC, Uvarovskii A, Ross TM, Guzmán CA, Perelson AS, Meyer-Hermann M (2014). Effects of aging on influenza virus infection dynamics. J Virol.

[CR123] Hernandez-Vargas EA, Velasco-Hernandez JX (2020). In-host Mathematical Modelling of COVID-19 in Humans. Annu Rev Control.

[CR124] Hethcote HW (2000). The mathematics of infectious diseases. SIAM Rev.

[CR125] Hoffman BU (2020). Significant relaxation of SARC-COV-2-targeted non-pharmaceutical interventions may result in profound mortality: a New York state modeling study. PLoS ONE.

[CR126] Holland J (1975). Adaptation in natural and artificial systems.

[CR127] Holland JH (1992). Genetic algorithms. Sci Am.

[CR128] Hooke R, Jeeves TA (1961). Direct search solution of numerical and statistical problems. J ACM.

[CR129] Hosseini E, Ghafoor KZ, Sadiq AS, Guizani M, Emrouznejad A (2020). COVID-19 optimizer algorithm, modeling and controlling of coronavirus distribution process. IEEE J Biomed Health Inform.

[CR130] Huang L, Liao Q, Qiu R, Liang Y, Long Y (2021). Prediction-based analysis on power consumption gap under long-term emergency: a case in Chinaunder COVID-19. Appl Energy.

[CR131] Hussain K, Salleh MNM, Cheng S, Shi Y (2019). Metaheuristic research: a comprehensive survey. Artif Intell Rev.

[CR132] Ibrahim RA, Elaziz MA, Lu S (2018). Chaotic opposition-based grey-wolf optimization algorithm based on differential evolution and disruption operator for global optimization. Exp Syst Appl.

[CR133] Ibrahim RA, Ewees AA, Oliva D, Elaziz MA, Lu S (2019). Improved salp swarm algorithm based on particle swarm optimization for feature selection. J Ambient Intell Humaniz Comput.

[CR134] Indu J, Jain VK, Jain R (2018). Correlation feature selection based improved-binary particle swarm optimization for gene selection and cancer classification. Appl Soft Comput.

[CR135] Iorio AW, Li X (2006) Incorporating directional information within a differential evolution algorithm for multi-objective optimization. In: Proceedings of the genetic and evolutionary computation conference (GECCO’06), pp 691–697

[CR136] Islam MM, Karray F, Alhajj R, Zeng J (2021). A review on deep learning techniques for the diagnosis of novel coronavirus (COVID-19). IEEE Access.

[CR137] Issa M, Hassanien AE, Oliva D, Helmi A, Ziedan I, Alzohiary A (2018). ASCA-PSO: adaptive sine cosine optimization algorithm integrated with particle swarm for pairwise local sequence alignment. Exp Syst Appl.

[CR138] Issa M, Abd Elaziz M (2020). Analyzing COVID-19 virus based on enhanced fragmented biological local aligner using improved ions motion optimization algorithm. Appl Soft Comput.

[CR139] Jamart J (1992). Statistical tests in medical research. Acta Oncol.

[CR140] Jang JSR (1993). ANFIS: adaptive-network-based fuzzy inference system. IEEE Trans Syst Man Cybern.

[CR141] Javidy B, Hatamlou A, Mirjalili S (2015). Ions motion algorithm for solving optimization problems. Appl Soft Comput.

[CR142] Jeyanathan M, Afkhami S, Smaill F, Miller MS, Lichty BD, Xing Z (2020). Immunological considerations for COVID-19 vaccine strategies. Nat Rev Immunol.

[CR143] Jorge DCP, Rodrigues MS, Silva MS, Cardim LL, da Silva NB, Silveira IH, Silva VAF, Pereira FAC, de Azevedo AR, Amad AAS, Pinho STR, Andrade RFS, Ramos PIP, Oliveira JF (2020) Assessing the nationwide impact of COVID-19 mitigation policies on the transmission rate of SARS-COV-2 in Brazil. medRxiv preprint: 10.1101/2020.06.26.20140780

[CR144] Kabir MM, Shahjahan M, Murase K (2011). A new local search based hybrid genetic algorithm for feature selection. Neurocomputing.

[CR145] Kang Y, Gao S, Liang Y, Li M, Rao J, Kruse J (2020). Multiscale dynamic human mobility flow dataset in the U.S. during the COVID-19 epidemic. Sci Data.

[CR146] Karaboga D, Basturk B (2008). On the performance of artificial bee colony (ABC) algorithm. Appl Soft Comput.

[CR147] Karakonstantis I, Vlachos A (2020). Bat algorithm applied to continuous constrained optimization problems. J Inf Optim Sci.

[CR148] Kennedy J, Eberhart R (1995) Particle swarm optimization. In: Proceedings of ICNN’95-international conference on neural networks, vol 4, pp 1942–1948, IEEE

[CR149] Kennedy J, Eberhart RC (1997) A discrete binary version of the particle swarm algorithm. In: Computational cybernetics simulation 1997, IEEE, vol 5, pp 4104–4108

[CR150] Kergassner A, Burkhardt C, Lippold D, Kergassner M, Pflug L, Budday D, Steinmann P, Budday S (2020). Memory-basedmeso-scalemodeling of Covid-19. Comput Mech.

[CR151] Kerschke P, Hoos HH, Neumann F, Trautmann H (2019). Automated algorithm selection: survey and perspectives. Evol Comput.

[CR152] Khan I, Shah D, Shah SS (2020). COVID-19 pandemic and its positive impacts on the environment: an updated review. Int J Environ Sci Technol.

[CR153] Khare P, Burse K (2016). Feature selection using genetic algorithm and classification using weka for Ovarian Cancer. Int J Comput Sci Inf Technol.

[CR154] Kim Y, Street WN, Menczer F (2000) Feature selection in unsupervised learning via evolutionary search. In: Proceedings of the sixth ACM SIGKDD international conference on knowledge discovery and data mining, pp 365–369

[CR155] Koziel S, Michalewicz Z (1999). Evolutionary algorithms, homomorphous mappings, and constrained parameter optimization. Evol Comput.

[CR156] Krivorot’ko OI, Kabanikhin SI, Zyat’kov NY, Prikhod’ko AY, Prokhoshin NM, Shishlenin MA (2020). Mathematical modeling and forecasting of COVID-19 in Moscow and Novosibirsk region. Numer Anal Appl.

[CR157] Lampinen J (2002) A constraint handling approach for the differential evolution algorithm. In: Proceedings of the 2002 congress on evolutionary computation (02TH8600), vol 2, IEEE, pp 1468–1473

[CR158] LeCun Y, Bengio Y, Hinton G (2015). Deep learning. Nature.

[CR159] Le Quere C, Jackson RB, Jones MW, Smith AJP, Abernethy S, Andrew RM, De-Gol AJ, Willis DR, Shan Y, Candell JG, Friedligstein P, Creutzig F, Peters GP (2020). Temporary reduction in daily global CO_2_ emissions during the COVID-19 forced confinement. Nat Clim Change.

[CR160] Li L, Yang Z, Dang Z, Meng C, Huang J, Meng H, Wang D, Chen G, Zhang J, Peng H, Shao Y (2020). Propagation analysis and prediction of the COVID-19. Infect Dis Model.

[CR161] Li S, Chen H, Wang M, Heidari AA, Mirjalili S (2020). Slime mould algorithm: a new method for stochastic optimization. Futur Gener Comput Syst.

[CR162] Li H, Liu Z, Ge J (2020). Scientific research progress of COVID-19/SARS-CoV-2 in the first five months. J Cell Mol Med.

[CR163] Liang JJ, Qin AK, Suganthan P, Baskar S (2006). Comprehensive learning particle swarm optimizer for global optimization of multimodal functions. IEEE Trans Evol Comput.

[CR164] Liang JJ, Qu BY, Suganthan PN, Hernández-Díaz AG (2013) Problem definitions and evaluation criteria for the CEC 2013 special session and competition on real-parameter optimization. Technical Report 201212, Computational Intelligence Laboratory, ZhengzhouUniversity, ZhengzhouChina and Nanyang Technological University, Singapore

[CR165] Libotte GB, Lobato FS, Platt GM, Silva Neto AJ (2020). Determination of an optimal control strategy for vaccine administration in COVID-19 pandemic treatment. Comput Methods Prog Biomed.

[CR166] Liu J, West M (2001) Combined parameter and state estimation in simulation-based filtering, in: sequential monte carlo methods in practice. Springer, pp 197–223

[CR167] Liu Z, Li Z, Chen W, Zhao Y, Yue H, Wu Z (2020). Path optimization of medical waste transport routes in the emergent public health event of COVID-19: a hybrid optimization algorithm based on the immune-ant colony algorithm. Int J Environ Res Public Health.

[CR168] Lobato FS, Steffen JV (2011). A new multi-objective optimization algorithm based on differential evolution and neighborhood exploring evolution strategy. J Artif Intell Soft Comput Res.

[CR169] Lobato FS, Steffen JV (2013). Multi-objective optimization firefly algorithm applied to (bio)chemical engineering system design. Am J Appl Math Stat.

[CR170] Lobato FS, Libotte GB, Platt GM (2020). Identification of an epidemiological model to simulate the COVID-19 epidemic using robust multiobjective optimization and stochastic fractal search. Comput Math Methods Med.

[CR171] Lu H, Ma X, Ma M (2021). A hybrid multi-objective optimizer-based model for daily electricity demand prediction considering COVID-19. Energy.

[CR172] Luchi F, Krohlingb RA (2015). Differential evolution and nelder-mead for constrained non-linear integer optimization problems. Procedia Comput Sci.

[CR173] Ma H, Simon D (2011). Blended biogeography-based optimization for constrained optimization. Eng Appl Artif Intell.

[CR174] Ma H, Fei M, Ding Z, Jin J (2012) Biogeography-based optimization with ensemble of migration models for global numerical optimization. In: 2012 IEEE congress on evolutionary computation. 10.1109/CEC.2012.6252930

[CR175] Ma H, Simon D, Fei M, Shu X, Chen Z (2014). Hybrid biogeographybased evolutionary algorithms. Eng Appl Artif Intell.

[CR176] Machova K, Mikula M, Gao X, Mach M (2020). Lexicon-based sentiment analysis using the particle swarm optimization. Electronics.

[CR177] Makade RG, Chakrabarti S, Jamil B (2020). Real-time estimation and prediction of the mortality caused due to COVID-19 using particle swarm optimization and finding the most influential parameter. Infect Dis Model.

[CR178] Mallipeddi R, Suganthan PN (2008) Empirical study on the effect of population size on Differential Evolution algorithm. In: Proceedings of IEEE congress on evolutionary computation, Hong Kong

[CR179] Mandal I, Pal S (2020). COVID-19 pandemic persuaded lockdown effects on environment over stone quarrying and crushing areas. Sci Total Environ.

[CR180] Marini F, Walczak B (2015). Particle swarm optimization (PSO) a tutorial. Chemom Intell Lab Syst.

[CR181] Martinez-Alvarez F, Asencio-Cortes G, Torres JF, Gutierrez-Aviles D, Melgar-Garcia L, Perez-Chacon R, Rubio-Escudero C, Riquelme JC, Troncoso A (2020). Coronavirus optimization algorithm: a bioinspired metaheuristic based on the COVID-19 propagation model. Big Data.

[CR182] McKee M, Stucker D (2020). If the world fails to protect the economy, COVID-19 will damage health not just now but also in the future. Nat Med.

[CR183] Medjahed SA, Ouali M (2020). Automatic system for COVID-19 diagnosis. Comput Sistemas.

[CR184] Mei X, Lee HC, Diao KY, Huang M, Lin B, Liu C, Xie Z, Ma Y, Robson PM, Chung M, Bernheim A, Mani V, Calcagno C, Li K, Li S, Shan H, Lv J, Zhao T, Xia J, Long Q, Steinberger S, Jacobi A, Deyer T, Luksza M, Liu F, Little BP, Fayad ZA, Yang Y (2020). Artificial intelligence-enabled rapid diagnosis of patients with COVID-19. Nat Med.

[CR185] Mersmann O, Preuss M, Trautmann H, Bischl B, Weihs C (2015). Analyzing the BBOB results by means of benchmarking concepts. Evol Comput.

[CR186] Miralles-Pechuan L, Jimenez F, Ponce H, Martinez-Villasenor L (2020) A deep Q-learning/genetic algorithms based novel methodology for optimizing COVID-19 pandemic government actions. arXiv:2005.07656v1

[CR187] Miranda L (2018). Pyswarms: a research toolkit for particle swarm optimization in python. J Open Source Softw.

[CR188] Mirjalili S, Mirjalili SM, Lewis A (2014). Grey wolf optimizer. Adv Eng Softw.

[CR189] Mirjalili S (2015). Moth-fame optimization algorithm: a novel nature inspired heuristic paradigm. Knowl Based Syst.

[CR190] Mirjalili S (2016). Dragonfly algorithm: a new meta-heuristic optimization technique for solving single-objective, discrete and multi-objective problems. Neural Comput Appl.

[CR191] Mirjalili S (2016). SCA: a sine cosine algorithm for solving optimization problems. Knowl-Based Syst.

[CR192] Mirjalili S, Lewis A (2016). The whale optimization algorithm. Adv Eng Softw.

[CR193] Mirjalili S, Mirjalili SM, Hatamlou A (2016). Multi-verse optimizer: a nature-inspired algorithm for global optimization. Neural Comput.

[CR194] Mirjalili S, Saremi S, Mirjalili SM, Coelho LDS (2016). Multi-objective grey wolf optimizer: a novel algorithm for multi-criterion optimization. Exp Syst Appl.

[CR195] Mirjalili SZ, Mirjalili S, Saremi S, Faris H, Aljarah I (2018). Grasshopper optimization algorithm for multi-objective optimization problems. Appl Intell.

[CR196] Mishra S, Rathee DS, Satapathy S, Mohanty RC, Gopi Krishna T, Chauhan RS (2020). Deep CNN-WCA and FLICM image segmentation for automatic detection and classification of COVID-19 diseases. PalArch’s J Archaeol Egipt Egyptol.

[CR197] Mohammed SN, Alkinani FS, Hassan YA (2020). Automatic computer aided diagnostic for COVID-19 based on chest x-ray image and particle swarm intelligence. Int J Intell Eng Syst.

[CR198] Moosavi SHS, Bardsiri VK (2017). Satin bowerbird optimizer. Eng Appl Artif Intell.

[CR199] Mouhoub M, Wang Z (2006) Ant colony with stochastic local search for the quadratic assignment problem. In: 2006 18th IEEE international conference on tools with artificial intelligence, IEEE, Arlington, VA, USA, pp 127–131

[CR200] Muhlenbein H, Schlierkamp-Voosen D (1993). Predictive models for the breeder genetic algorithm I. Continuous parameter optimization. Evolut Comput.

[CR201] Muhlenbein H, Mahnig T (1999). FDA-A scalable evolutionary algorithm for the optimization of additively decomposed functions. Evol Comput.

[CR202] Mustopa A, Hermanto, Anna, Pratama EB, Hendini A, Risdiansyah D (2020) Analysis of user reviews for the PeduliLindungi application on Google Play using support vector machine and naïve Bayes algorithm based on particle swarm optimization. In: 2020 Fifth international conference on informatics computing (ICIC), IEEE. 10.1109/ICIC50835.2020.9288655

[CR203] Naraigh LO, Byrne A (2020). Piecewise-constant optimal control strategies for controlling the outbreak of COVID-19 in the Irish population. Math Biosci.

[CR334] Nelder JA, Mead R (1965) A simplex method for function minimization. Comput J 7:308-313

[CR204] Neri F, Tirronen V (2010). Recent advances in differential evolution: a survey and experimental analysis. Artif Intelli Gence Rev.

[CR205] Ngie HM, Nderu L, Mwigeriri DG (2020) Tree-based regressor ensemble for viral infectious diseases spread prediction. ceur-ws.org/Vol-2689/paper12

[CR206] Niazi MUB, Kibangou A, Canduas-de-Wit C, Nikitin D, Tumash L, Bliman PA (2020) Modeling and control of COVID-19 epidemic through testing policies. arxiv:2010.15438v1 10.1016/j.arcontrol.2021.09.004 PMC8514419 34664008

[CR207] Nowakova J, Kromer P, Platos J, Snasel V (2020) Preprocessing COVID-19 radiographic images by evolutionary column subset selection. In: Advances in intelligent networking and collaborative systems, advances in intelligent systems and computing 1263, Springer, pp 425–436

[CR208] Nowakowska J, Sobocinska J, Lewicki M, Lemanska Z, Rzymski P (2020). When science goes viral: the research response during three month of the COVID-19 outbreak. Biomed Pharmacother.

[CR209] Oliveira JF, Jorge DCP, Veiga RV, Rodrigues MS, Torquato MF, da Silva NB, Fiaconne RL, Castro CP, Paiva ASS, Cardim LL, Amad AAS, Lima EBAF, Souza DS, Pinho STR, Ramos PIP, Andrade RFS (2021). Mathematical modeling of COVID-19 in 14.8 million individuals in Bahia, Brasil. Nat Commun.

[CR210] Opara KR, Arabas J (2019). Differential evolution: a survey of theoretical analyses. Swarm Evol Comput.

[CR211] Ozsahin I, Sekeroglu B, Musa MS, Mustapha MT, Ozsahin DU (2020) Review on diagnosis of COVID-19 from chest CT images using artificial intelligence. Comput Math Methods Med 9756518 10.1155/2020/9756518 PMC7519983 33014121

[CR212] Pace F, Santilano A, Godio A (2019). Particle swarm optimization of 2D magnetotelluric data. Geophysics.

[CR213] Paggi M (2020). An analysis of the Italian lockdown in retrospective using particle swarm optimization in machine learning applied to an epidemiological model. Physics.

[CR214] Paggi M (2020b) Simulation of Covid-19 epidemic evolution: are compartmental models really predictive? arXiv:2004.08207

[CR215] Pan JS, Nguyen TT, Hu SC, Dao TK, Ngo TG (2019). Diversity enhanced ion motion optimization for localization in wireless sensor network. J Inf Hiding Multimed Signal Process.

[CR216] Pawar SN, Bichkar RS (2015). Genetic algorithm with variable length chromosomes for network intrusion detection. Int J Autom Comput.

[CR217] Peng T, Huanchen W, Dongme Z (1996). Simulated annealing for the quadratic assignment problem: a further study. Comput Ind Eng.

[CR218] Peng Y, Sun K, He S, Peng D (2019). Parameter identification of fractional-order discrete chaotic systems. Entropy.

[CR219] Pham DT, Ghanbarzadeh A, Koc E, Otri S, Rahim S, Zaidi M (2005) The bees algorithm. Technical Note, Manufacturing Engineering Centre, Cardiff University, UK

[CR220] Pinter G, Felde I, Mosavi A, Ghamisi P, Gloaguen R (2020). COVID-19 pandemic prediction for Hungary; a hybrid machine learning approach. Mathematics.

[CR221] Piotrowski AP (2017). Review of differential evolution population size. Swarm Evol Comput.

[CR222] Piotrowski AP, Napiorkowski MJ, Napiorkowski JJ, Rowinski PM (2017). Swarm intelligence and evolutionary algorithms: performance versus speed. Inf Sci.

[CR223] Piotrowski AP, Napiorkowski JJ (2018). Step-by-step improvement of JADE and SHADE-based algorithms: success or failure?. Swarm Evol Comput.

[CR224] Piotrowski AP, Napiorkowski JJ, Piotrowska AP (2020). Population size in particle swarm optimization. Swarm Evolut Comput.

[CR225] Poli R, Kennedy J, Blackwell T (2007). Particle swarm optimization. Swarm Intell.

[CR226] Price KV, Awad NH, Ali MZ, Suganthan PN (2019) The 2019 100-digit challenge on real-parameter, single-objective optimization: analysis of results. Nanyang Technological University, Singapore, Technical Report. http://www.ntu.edu.sg/home/epnsugan

[CR227] Punitha S, Al-Turjman F, Stephan T (2020) Genetically optimized computer-aided diagnosis for detection and classification of COVID-19. In: AI-Powered IoT for COVID-19, ed. Fadi Al Turjman, CRC Press, Boca Raton, FL, USA

[CR228] Qiao S, Zhou Y, Zhou Y, Wang R (2018). A simple water cycle algorithm with percolation operator for clustering analysis. Soft Comput.

[CR229] Quaranta G, Formica G, Machado JT, Lacarbonara W, Masri SF (2020). Understanding COVID-19 nonlinear multi-scale dynamic spreading in Italy. Nonlinear Dyn.

[CR230] Rahimi I, Chen F, Gandomi AH (2021). A review on COVID-19 forecasting models. Neural Comput Appl.

[CR231] Rahmandad H, Lim TY, Sterman J (2020) Estimating COVID-19 under-reporting across 86 nations: implications for projections and control. medRxiv preprint. 10.1101/2020.06.24.20139451

[CR232] Rakshit P, Konar A (2015). Differential evolution for noisy multiobjective optimization. Artif Intell.

[CR233] Rashedi E, Nezamabadi-pour H, Saryazdi S (2009). GSA: a gravitational search algorithm. Inf Sci.

[CR234] Rasheed J, Jamil A, Hameed AA, Aftab U, Aftab J, Shah SA, Draheim D (2020). A survey on artificial intelligence approaches in supporting frontline workers and decision makers for the COVID-19 pandemic. Chaos Solitons Fract.

[CR235] Rashid MT, Wang D (2020). CovidSens: a vision on reliable social sensing for COVID-19. Artif Intell Rev.

[CR236] Ratnaweera A, Halgamuge SK, Watson HC (2004). Self-organizing hierarchical particle swarm optimizer with time-varying acceleration coefficients. IEEE Trans Evol Comput.

[CR237] Rica S, Ruz GA (2020) Estimating SIR model parameters from data using differential evolution: an application with COVID-19 data. In: 2020 IEEE conference on computational intelligence and bioinformatics biology (CIBCB), Vua del Mar, Chile. 10.1109/CIBCB48159.2020.9277708

[CR238] Ricardo CLA, Hernandez-Vargas EA (2020) The risk of lifting COVID-19 confinement in Mexico. medRxiv 10.1101/2020.05.28.20115063

[CR239] Roberts M, Driggs D, Thorpe M, Gilbey J, Yeung M, Ursprung S, Aviles-Rivero AI, Etmann C, McCague C, Beer L, Weir-McCall JR, Teng Z, Rudd JHF, Sala E, Schönlieb CB (2020) Machine learning for COVID-19 detection and prognostication using chest radiographs and CT scans: a systematic methodological review. arXiv.2008.06388v3

[CR240] Ronkkonen J, Kukkonen S, Price KV (2005) Real parameter optimization with differential evolution. In: Proceedings of the IEEE congress on evolutionary computation, pp 506–513

[CR241] Sahlol AT, Yousri D, Ewees AA, Al-qaness MAA, Damasevicius R, Abd Elaziz M (2020). COVID-19 image classification using deep features and fractional order marine predators algorithm. Sci Rep.

[CR242] Saif S, Das P, Biswas S (2021). A hybrid model based on mBA-ANFIS for COVID 19 confirmed cases prediction and forecast. J Inst Eng India Ser B.

[CR243] Sainz-Pardo JL, Valero J (2020) COVID-19 and other viruses: holding back its expansion by massive testing. arxiv:2012.12345v1 10.1016/j.eswa.2021.115710 PMC8351357 34393387

[CR244] Salimi H (2015). Stochastic fractal search: a powerful metaheuristic algorithm. Knowl-Based Syst.

[CR245] Sallam KM, Elsayed SM, Chakrabortty RK, Ryan MJ (2020) Improved multi-operator differential evolution algorithm for solving unconstrained problems. In: 2020 IEEE congress on evolutionary computation (CEC), IEEE, pp1–8

[CR246] Samal NR, Konar A, Das S, Abraham A (2007) A closed loop stability analysis and parameter selection of the particle swarm optimization dynamics for faster convergence. In: Proceedings of the IEEE international conference on evolutionary computation (CEC), IEEE Press, pp 1769–1776

[CR247] Sanche S, Lin YT, Xu C, Romero-Sevenson E, Hengartner NW, Ke R (2020) The novel coronavirus, 2019-nCoV, is highly contagious and more infections than initially estimated. arxiv:2002.03268

[CR248] Santucci V, Baioletti M, Milani A (2016). Algebraic differential evolution algorithm for the permutation flowshop scheduling problem with total flowtime criterion. IEEE Trans Evol Comput.

[CR249] Saqib Nawaz M, Fournier-Viger P, Shojaee A, Fujita H (2021). Using artificial intelligence techniques for COVID-19 genome analysis. Appl Intell.

[CR250] Sarkar S, Das S, Chaudhuri SS (2016). Hyper-spectral image segmentation using Renyi entropy based multi-level thresholding aided with differential evolution. Exp Syst Appl.

[CR251] Satapathy SC, Hemanth DJ, Kadry S, Manogaran G, Hannon NMS, Rajinikanth V (2020) Segmentation and evaluation of COVID-19 lesion from CT scan Slices—a study with Kapur/Otsu function and cuckoo search algorithm. researechsquare.com

[CR252] Sayed GI, Khoriba G, Haggag MH (2018). A novel chaotic salp swarm algorithm for global optimization and feature selection. Appl Intell.

[CR253] Sazvar Z, Tanhaeean M, Aria SS, Akbari A, Ghaderi SF, Iranmanesh SH (2020). A computational intelligence approach to detect future trends of COVID-19 in France by analyzing Chinese data. Health Educ Health Promot.

[CR254] Senel FA, Gokçe F, Yuksel AS, Yigit T (2019). A novel hybrid PSO–GWO algorithm for optimization problems. Eng Comput.

[CR255] Shaban WM, Rabie AH, Saleh AI, Abo-Elsoud MA (2020). A new COVID-19 patients detection strategy (CPDS) based on hybrid feature selection and enhanced KNN classifier. Knowl Based Syst.

[CR256] Shao W, Pi D, Shao Z (2017). An extended teaching-learning based optimization algorithm for solving no-wait flow shop scheduling problem. Appl Soft Comput.

[CR257] Sharma P, Sharma H, Kumar S, Bansal JC (2019) A review of scale factor strategies in differential evolution algorithms. In: Soft computing for problem solving. Advances in intelligent systems and computing, vol 817, pp 925–943

[CR258] Sheybani Z, Dokoohaki MH, Negahdaripour M, Dehdashti M, Zolghadr H, Moghadami M, Masoompour SM, Zolghadr AR (2020) The role of folic acid in the management of respiratory disease caused by COVID-19. ChemRxiv 10.26434/chemrxiv.12034980.v1

[CR259] Shi Y, Eberhart RC (1998) Parameter selection in particle swarm optimization. In: Evolutionary programming VII, Proceedings, Naw York, Springer Verlag, pp 591–600

[CR260] Simon D (2008). Biogeography-based optimization. IEEE Trans Evol Comput.

[CR261] Singh D, Kumar V, Vaishali KM (2020). Classification of COVID-19 patients from chest CT images using multi-objective differential evolution–based convolutional neural networks. Eur J Clin Microbiol Infect Dis.

[CR262] Singh D, Kumar V, Yadav V, Kaur M (2020). Deep neural network-based screening model for COVID-19-infected patients using chest x-ray images. Int J Pattern Recognit Artif Intell.

[CR263] Sörensen K (2015). Metaheuristics—the metaphor exposed. Int Trans Oper Res.

[CR264] Souza DL, Lobato FS, Gedraite R (2015). Robust multiobjective optimization applied to optimal control problems using differential evolution. Chem Eng Technol.

[CR265] Storn R, Price KV (1995) Differential evolution—a simple and efficient adaptive scheme for global optimization over continuous spaces. Technical Report TR-95-012, International Computer Sciences Institute, Berkeley, California, USA

[CR266] Storn R, Price KV (1997). Differential evolution—a simple and efficient heuristic for global optimization over continuous spaces. J Glob Optim.

[CR267] Strasak AM, Zaman Q, Pfeiffer KP, Gobel G, Ulmer H (2007). Statistical errors in medical research—a review of common pitfalls. Swiss Med Wkly.

[CR268] Suresh K, Ghosh S, Kundu D, Sen A, Das S, Abraham A (2008) Inertia-adaptive particle swarm optimizer for improved global search. In: Eight international conference on intelligent systems design and applications (ISDA 2008), IEEE Press

[CR269] Suresh K, Kundu D, Shosh S, Das S, Abraham A, Han SY (2009). Multi-objective differential evolution for automatic clustering with applications to micro-array data analysis. Sensors.

[CR270] Susanto H, Leu FY, Caesarenda W, Ibrahim F, Haghi PK, Khusni U, Glowacz A (2020). Managing cloud intelligent systems over digital ecosystems: revealing emerging app technology in the time of the COVID-19 pandemic. Appl Syst Innov.

[CR271] Syeda HB, Syed M, Sexton K, Syed S, Begum S, Syed F, Yu F (2020) The role of machine learning techniques to tackle COVID-19 crisis: a systematic review. medRxiv preprint. 10.1101/2020.08.23.20180158

[CR272] Szeto WY, Wu Y, Ho SC (2011). An artificial bee colony algorithm for the capacitated vehicle routing problem. Eur J Oper Res.

[CR273] Tanabe R, Fukunaga A (2014) Improving the search performance of SHADE using linear population size reduction. In: Proceedings of the IEEE congress on evolutionary computation, Bejing, China, pp 1658–1665

[CR274] Ter Braak CJF (2006). A Markov Chain Monte Carlo version of the genetic algorithm differential evolution: easy Bayesian computing for real parameter spaces. Stat Comput.

[CR275] Ter Braak CJF, Vrugt JA (2008). Differential evolution Markov Chain with snooker updater and fewer chains. Stat Comput.

[CR276] Therib MA, Al-Baghdadi AF, Marzog HA (2020). Medical remotely caring with COVID-19 virus infected people using optimized wireless are tracing system. TELKOMNIKA Telecommun Comput Electron Control.

[CR277] Thomsen R, Christensen MH (2006). MolDock: a new technique for high-accuracy molecular docking. J Med Chem.

[CR278] Too J, Abdullah A, Saad NM, Ali NM, Tee W (2018). A new competitive binary grey wolf optimizer to solve the feature selection problem in EMG signals classification. Computers.

[CR279] Too J, Abdullah A, Saad NM, Tee W (2019). EMG feature selection and classification using a Pbest-guide binary particle swarm optimization. Computation.

[CR280] Too J, Mirjalili S (2020). A hyper learning binary dragonfly algorithm for feature selection: a COVID-19 case study. Knowl-Based Syst.

[CR281] Tseng VS, Ying JJC, Wong STC, Cook DJ, Liu J (2020). Computational intelligence techniques for combating COVID-19: a survey. IEEE Comput Intell Mag.

[CR282] Tsutsui S, Ghosh A (1997). Genetic algorithms with a robust solution searching scheme. IEEE Trans Evol Comput.

[CR283] Unlu E, Leger H, Motornyi O, Rukubayihunga A, Ishacian T, Chouiten M (2020) Epidemic analysis of COVID-19 outbreak and counter-measures in France. medRxiv preprint 10.1101/2020.04.27.20079962

[CR284] van Laarhoven PJ, Aarts EH (1987) Simulated annealing. In: Simulated annealing: theory and applications. Springer, Dordrecht, pp 7–15

[CR285] Van Tinh N (2020). Forecasting of COVID-19 confirmed cases in Vietnam using fuzzy time series model combined with particle swarm optimization. Comput Res Prog Appl Sci Eng.

[CR286] Van Tinh N (2020). Forecasting for coronavirus disease spread in Vietnam using fuzzy time series model and particle swarm optimization. Int Multiling J Sci Technol.

[CR287] Vecek N, Mernik M, Crepinsek M (2014). A chess rating system for evolutionary algorithms: a new metod for the comparison and ranking of evolutionary algorithms. Inf Sci.

[CR288] Vrbancic G, Zorman M, Podgorelec V (2019) Transfer learning tuning utilizing grey wolf optimizer for identification of brain hemorrhage from head ct images. In StuCoSReC: proceedings of the 2019 6th student computer science research conference, pp 61–66

[CR289] Vrbancic G, Pecnik S, Podgorelec V (2020) Identification of COVID-19 X-ray images using CNN with optimized tuning of transfer learning. In: 2020 International conference on innovations in intelligent systems and applications (INISTA), Novi Sad, Srbia, IEEE. 10.1109/INISTA49547.2020.9194615

[CR290] Vrugt J, ter Braak C, Gupta H, Robinson B (2009). Accelerating Markov chain Monte Carlo simulation by differential evolution with self-adaptive randomized subspace sampling. Int J Nonlinear Sci Numer Simul.

[CR291] Wang X, Yang J, Teng X, Xia W, Jensen R (2007). Feature selection based on rough sets and particle swarm optimization. Pattern Recogn Lett.

[CR292] Wang R, Hu G, Jiang C, Lu H, Zhang Y (2020a) Data analytics for the CQVID-19 Epidemic. In: 2020 IEEE 44th annual computers, software, and applications conference (COMPSAC), pp 1261–1266

[CR293] Wang S, Jia H, Peng X (2020). Modified salp swarm algorithm based multilevel thresholding for color image segmentation. Math Biosci Eng.

[CR294] Weber M, Neri F, Tirronen V (2013). A study on scale factor/crossover interaction in distributed differential evolution. Artif Intell Rev.

[CR295] Weynants L, Van Calster B, Collins GS, Riley RD, Heinze G, Schuit E, Bonten MMJ, Dahly DL, Damen JAA, Debray TPA, de Jong VMT, De Vos M, Dhiman P, Haller MC, Harhay MO, Henckaerts L, Heus P, Kreuzberger N, Lohmann A, Luijken K, Ma J, Martin GP, Navarro CLA, Reitsma JB, Sergeant JC, Shi C, Skoetz N, Smits LJM, Snell KIE, Sperrin M, Spijker R, Steyerberg EW, Takada T, Tzoulaki I, van Kuijk SMJ, van Royen FS, Verbake JY, Wallisch C, Wilkinson J, Wolff R, Hooft L, Moons KGM, van Smeden M (2020). Prediction models for diagnosis and prognosis of covid-19: systematic review and critical appraisal. BMJ.

[CR296] Whitley D, Starkweather T, Bogart C (1990). Genetic algorithms and neural networks: optimizing connections and connectivity. Parallel Comput.

[CR297] Whitley D (1994). A genetic algorithm tutorial. Stat Comput.

[CR298] Woldesenbet YG, Yen GG, Tessema BG (2009). Constraint handling in multiobjective evolutionary optimization. IEEE Trans Evol Comput.

[CR299] Wong GN, Weiner ZJ, Tkachenko AV, Elbanna A, Maslov S, Goldenfeld N (2020). Modeling COVID-19 dynamics in Illinois under non-pharmaceutical interventions. Phys Rev X.

[CR300] Wu CX, Liao MH, Karatas M, Chen SY, Zheng YJ (2020). Real-time neural network scheduling of emergency medical mask production during COVID-19. Appl Soft Comput.

[CR301] Xavier MP, Reis RF, dos Santos RW, Lobosco M (2020) A simplified model of the human immune system response to COVID-19. In: 2020 IEEE international conference on bioinformatics and biomedicine (BIBM), Soeul, South Korea, pp 1311–1317

[CR302] Xin B, Chen J, Zhang J, Fang H, Peng ZH (2012). Hybridizing differential evolution and particle swarm optimization to design powerful optimizers: a review and taxonomy. IEEE Trans Syst Man Cybern Part C Appl Rev.

[CR303] Xing H, Zhou X, Wang X, Luo S, Dai P, Li K, Yang H (2019). An integer encoding grey wolf optimizer for virtual network function placement. Appl Soft Comput.

[CR304] Yang XS (2008). Nature-inspired metaheuristic algorithms.

[CR305] Yang XS, Deb S (2009) Cuckoo search via Lévy Fights. In: Proceedings of the world congress. Nature biologically inspired comput. (NaBIC), pp 210–214

[CR306] Yang XS (2012) Flower pollination algorithm for global optimization. In: International conference on unconventional computing and natural computation. Springer, Berlin, Germany, pp 240–249

[CR307] Yang CH, Wu KC, Lin YS, Chuang LY, Chang HW (2018). Protein folding prediction in the HP model using ions motion optimization with a greedy algorithm. BioData Min.

[CR308] Yang GZ, Nelson BJ, Murphy RR, Choset H, Christensen H, Collins SH, Dario P, Goldberg K, Ikuta K, Jacobstein N, Kragic D, Taylor RH, McNutt M (2020). Combating COVID-19—the role of robotics in managing public health and infectious diseases. Sci Robot.

[CR309] Yao X, Han J (2020) COVID-19 detection via wavelet entropy and biogeography-based optimization. In: COVID-19: prediction, decision-making, and its impacts. Springer, pp 69–76

[CR310] Yousefpour A, Jahanshahi H, Bekiros S (2020). Optimal policies for control of the novel coronavirus disease (COVID-19) outbreak. Chaos Solitons Fract.

[CR311] Yousri D, Mirjalili S (2020). Fractional order Cuckoo Search algorithm for parameter identification of the fractional-order chaotic, chaotic with noise and hyper-chaotic financial systems. Eng Appl Artif Intell.

[CR312] Yousri D, Elaziz MA, Abualigah L, Oliva D, Al-qaness MAA, Ewees AA (2021). COVID-19 x-ray images classification based on enhanced fractional-order cuckoo search optimizer using heavy-tailed distributions. Appl Soft Comput.

[CR313] Yue CT, Price KV, Suganthan PN, Liang JJ, Ali MZ, Qu BY, Awad NH, Biswas PP (2019) Problem definitions and evaluation criteria for the CEC 2020 special session and competition on single objective bound constrained numerical optimization. Technical Report 201911, Computational Intelligence Laboratory, Zhengzhou University, China and Nanyang Technological University, Singapore

[CR314] Zaharie D (2009). Influence of crossover on the behavior of differential evolution algorithms. Appl Soft Comput.

[CR315] Zamuda A, Brest J, Boskovic B, Zumer V (2007) Differential evolution for multiobjective optimization with self adaptation. In: IEEE congress on evolutionary computation. Singapore: IEEE, pp 3617–3624

[CR316] Zamuda A, Brest J, Boskovic B, Zumer V (2009) Differential evolution with self-adaptation and local search for constrained multiobjective optimization. In: IEEE congress on evolutionary computation. Trondheim, Norway: IEEE, pp 195–202

[CR317] Zhan C, Zheng Y, Lai Z, Hao T, Li B (2020). Identifying epidemic spreading dynamics of COVID-19 by pseudocoevolutionary simulated annealing optimizers. Neural Comput Appl.

[CR318] Zhang Q, Li H (2007). MOEA/D: a multiobjective evolutionary algorithm based on decomposition. IEEE Trans Evol Comput.

[CR319] Zhang J, Sanderson ZC (2009). JADE: adaptive differential evolution with optional external archive. IEEE Trans Evol Comput.

[CR320] Zhang Y, Wang SH, Ji GL, Dong ZC (2013) An MR brain images classifier system via particle swarm optimization and kernel support vector machine. Sci World J 130134 10.1155/2013/130134 PMC3791634 24163610

[CR321] Zhao F, Zhang J, Wang J, Zhang C (2015). A shuffled complex evolution algorithm with opposition-based learning for a permutation flow shop scheduling problem. Int J Comput Integr Manuf.

[CR322] Zhao J, Tang D, Liu Z, Cai Y, Dong S (2019). Spherical search optimizer: a simple yet efficient meta-heuristic approach. Neural Comput Appl.

[CR323] Zhao W, Zhang Z, Wang L (2020). Manta Ray foraging optimization: an effective bio-inspired optimizer for engineering optimizations. Eng Appl Artif Intell.

[CR324] Zheng Y, Ling H, Xue J, Chen S (2014). Population classification in fire evacuation: a multiobjective particle swarm optimization approach. IEEE Trans Evol Comput.

[CR325] Zheng YJ, Ling HF, Xue JY (2014). Ecogeography-based optimization: enhancing biogeography-based optimization with ecogeographic barriers and differentiations. Comput Oper Res.

[CR326] Zheng YJ (2015). Wave water optimization: a new nature inspired metaheuristic. Comput Oper Res.

[CR327] Zheng YJ, Lu XQ, Du YC, Xue Y, Sheng WG (2019). Water wave optimization for combinatorial optimization: Design strategies and applications. Appl Soft Comput.

[CR328] Zheng YJ, Yu SL, Yang JC, Gan TE, Song Q, Yang J, Karatas M (2020a) Intelligent optimization of diversified community prevention of COVID-19 using traditional Chinese medicine. arXiv:2007.13926v1

[CR329] Zheng YJ, Chen X, Gan TE, Zhang MX, Sheng WG, Wang L (2020b) Balancing common treatment and epidemic control in medical procurement during COVID-19: transform-and-divide evolutionary optimization. arXiv:2008.00395v1

[CR330] Zheng YJ, Yu SL, Yang JC, Gan TE, Song Q, Yang J, Karatas M (2020). Intelligent optimization of diversified community prevention of COVID-19 using traditional Chinese medicine. IEEE Comput Intell Mag.

[CR331] Zorarpaci E, Ozel SA (2016). A hybrid approach of differential evolution and artificial bee colony for feature selection. Exp Syst Appl.

[CR332] Zou X, Fang Z, Xiong S (2020). A discrete particle swarm optimization method for assignment of supermarket resources to urban residential communities under the situation of epidemic control. Appl Soft Comput J.

[CR333] Zreiq R, Kamel S, Boubaker S, Al-Shammary AA, Algahtani FD, Alshammari F (2020). Generalized Richards model for predicting COVID-19 dynamics in Saudi Arabia based on particle swarm optimization algorithm. AIMS Public Health.

